# Tunable polaritonic topologies generated by non-local photonic modes

**DOI:** 10.1038/s41565-026-02174-5

**Published:** 2026-05-12

**Authors:** Enrico Baù, Connor Heimig, Jonas Biechteler, Florian Mangold, Julian Schwab, Manobina Karmakar, Leonardo Menezes, Haoran Ren, Stefan A. Maier, Harald Giessen, Andreas Tittl

**Affiliations:** 1 Chair in Hybrid Nanosystems, Nano-Institute Munich, Department of Physics, LMU, Munich, Germany; 2 4th Physics Institute, Research Center SCoPE, and Integrated Quantum Science and Technology Center, University of Stuttgart, Stuttgart, Germany; 3 Departamento de Física, Universidade Federal de Pernambuco, Recife, Brazil; 4 School of Physics and Astronomy, Monash University, Clayton, Victoria Australia; 5 Department of Physics, Imperial College London, London, UK; 6 Institute of Photonics, Hamburg University of Technology, Hamburg, Germany

**Keywords:** Nanophotonics and plasmonics, Two-dimensional materials, Polaritons, Sub-wavelength optics, Silicon photonics

## Abstract

Photonic skyrmions are topological textures that exhibit remarkable resilience to environmental perturbations and support deeply subwavelength features, making them promising candidates for high-resolution microscopy, optical computing devices and ultrahigh-density information encoding. However, in contrast to free-space optical skyrmions, all existing approaches to generate polaritonic field skyrmions are limited by a lack of dynamic tunability. In general, without engineering the phase of the incident light, both their lattice site diameter and total topological charges remain fixed after fabrication. These constraints originate from a shared reliance on wavelength-dependent coupling structures or complex excitation conditions. To overcome these limitations, we introduce the concept of dynamically controllable polaritonic topologies generated by non-local photonic modes. Here we leverage quasi-bound states in the continuum resonances in dielectric metasurfaces to launch hyperbolic phonon polaritons in hexagonal boron nitride that interfere to create highly confined photonic skyrmion lattices with diameters down to 271 nm (*λ*/25). Thanks to the steep dispersion of hexagonal boron nitride, we can change the excitation frequency to achieve control over the size of individual photonic skyrmions within the same physical resonator structure. In addition, our platform is not limited to one type of topology but can generate optical meron lattices and *kπ*-twist skyrmions through straightforward variations in resonator shape, providing a feasible path towards skyrmion multiplexing and near-arbitrary topologies. The synergistic integration of resonant metasurfaces with polaritonic topologies has potential applications for nanophotonics, such as topological lasing, nonlinear optics and twistronics, as well as for condensed matter physics, such as Chern insulators and topological edge states.

## Abstract (web summary)

Tunable polaritonic skyrmions and related topological textures are generated by non-local photonic metasurfaces, enabling reconfigurable nanoscale topologies on a chip.

## Main

Topology provides a foundational framework for understanding a wide range of natural phenomena^1–3^. Among its key manifestations are topological defects, which cannot be removed or transformed without fundamentally altering the system’s configuration, intrinsically preventing their decay. The skyrmion^4^ is a prime example, consisting of a three-dimensional (3D) vector field mapped onto a two-dimensional (2D) plane. It is typically described as a vector field encoding distinct mappings on a 3D unit sphere in order-parameter space, capturing the winding and twisting of the field. A skyrmion is characterized by fully covering the unit sphere such that all possible orientations of the vector field are represented. In condensed matter and solid-state physics, skyrmions appear in systems ranging from magnetic materials^5,6^ to superconductors^7,8^, superfluids^9^ and liquid crystals^10–12^.

Recently, topological defects have been extended to photonics, where skyrmions were observed via controlled interference of free-space waves^13–16^ and surface plasmon polaritons^17–19^. These photonic skyrmions exhibit deeply subwavelength features^20^ and inherent topological robustness against material defects and environmental perturbations^21–23^, highlighting their potential for optical computing, metrology and twistronics^24^. They also possess non-trivial features such as topological domain walls, tunable via the ratio of in-plane to out-of-plane momentum. This enables transitions from bubble-type skyrmions with sharp domain walls to Néel-type skyrmions with smeared domain walls^17,25^. Following their initial realization in plasmonics, recent demonstrations include free-space skyrmions^13,26^, skyrmion bags^24^ and various polaritonic topologies, such as optical meron lattices^27^, and deeply subwavelength optical vortices carrying orbital angular momentum. The latter were achieved by interfering surface phonon polaritons in isotropic polar materials^28^ (*ε*_*xx*_ = *ε*_*yy*_ = *ε*_*zz*_) or hyperbolic phonon polaritons (HPhPs) in anisotropic polar materials^29,30^ (*ε*_*xx*_ = *ε*_*yy*_ ≠ *ε*_*zz*_), with applications in structured thermal emission^31^.

**Fig. 1:**
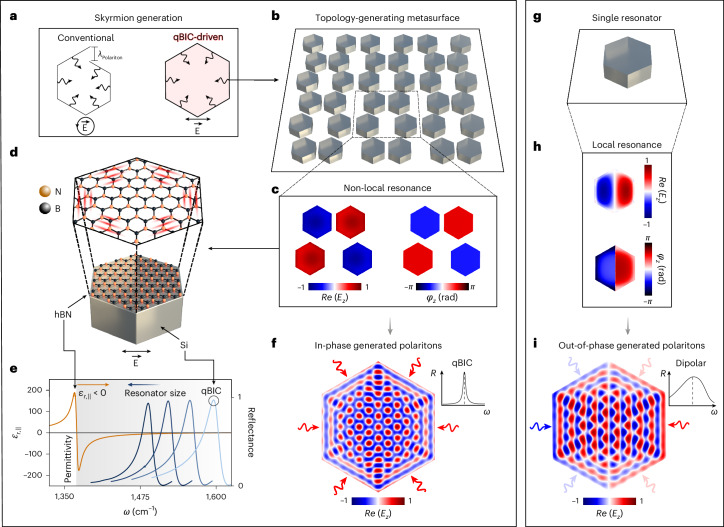
Non-local photonic mode-driven polaritonic topologies. **a**, A comparison between polaritonic topologies generated via conventionally used wavelength-dependent coupling structures^17,24^ (left) and our topology-generating metasurface (right). While previous platforms relied on circularly polarized incident light and polariton wavelength-dependent offsets to compensate for the phase mismatch at each edge, our approach enables the generation of HPhPs in regular polygons. **b**, An illustration of the topology-generating metasurface introduced in this work, consisting of hexagonal amorphous silicon resonators on a CaF_2_ substrate that supports the non-local qBIC resonance. **c**, The simulated real part *Re*(*E*_*z*_) (left) and phase *φ*_*z*_ (right) of the out-of-plane electric field at the qBIC resonance. The optical phase on the surface of each resonator is uniform and does not contain any singularities. **d**, A schematic of a dielectric resonator covered by hBN and illuminated with linearly polarized light. The excitation launches HPhPs at the edges of the resonator. **e**, The real part of the in-plane permittivity of hBN (orange curve) *ε*_*r*,*||*_ and reflectance spectra (blue curves) of the qBIC metasurface simulated for various resonator sizes, from smaller (light blue) to larger (dark blue). For the modelling of the permittivity of hBN and the calculated dispersion, see Supplementary Notes 1 and 2 and Supplementary Fig. 3, respectively. **f**, The qBIC resonances lie spectrally within the in-plane RS-band of hBN to excite HPhPs (grey shaded area in **e**), allowing for the in-phase generation of HPhPs at each resonator edge, resulting in photonic skyrmion lattices. **g**–**i**, Our approach contrasts with the use of local modes, such as a dipolar resonance in single resonators (**g**), which do not generate uniform field distributions (**h**) and therefore no notable topological configurations can be observed (**i**). Simulations of the out-of-plane electric fields were conducted at *ω* = 1,560 cm^−1^, within the RS-band of hBN.

However, existing approaches for generating polaritonic field skyrmions^14,25,32^ rely on non-reconfigurable, wavelength-dependent structures such as gratings or phase-correcting offsets, or on structured light such as radial polarization to launch and interfere surface waves (Fig. 1a, left). These constraints limit same-structure topological tunability, hindering integration into optical computing platforms requiring broadband reconfigurability. This contrasts with free-space skyrmions, where tunability has been demonstrated^13^. In addition, excitation typically requires circular or radial polarization^33^, necessitating extra optical elements such as waveplates and increasing experimental complexity.

In this work, we introduce structured polaritonic topologies generated through non-local photonic resonances^34,35^, enabling skyrmion formation without phase-correcting offsets and using linearly polarized light (Fig. 1a, right). We realize this by using arrays of high refractive-index dielectric hexagonal resonators (Fig. 1b) on a transparent CaF_2_ substrate supporting quasi-bound states in the continuum (qBICs)^36,37^ under linear polarization. These resonances arise from engineered in-plane asymmetry within each unit cell, allowing control over their linewidths^36^. Crucially, symmetry-protected qBICs require extended periodic arrays rather than isolated resonators^38,39^. This allows multiple resonators to be driven simultaneously by the same excitation, with topology governed by resonator geometry. As a result, optical skyrmions can be scaled from single structures to photonic chips, enabling large-area metasurfaces with multiple encoded skyrmion lattices.

On the basis of this principle, we experimentally demonstrate the generation and reconfigurability of qBIC-driven polaritonic topologies via interference of HPhPs in hexagonal boron nitride (hBN) thin films. Using scattering-type scanning near-field optical microscopy (s-SNOM), we resolve amplitude and phase of deeply subwavelength photonic skyrmion lattices induced by the non-local qBIC. By tuning the excitation frequency within the Reststrahlen (RS) band of hBN, we dynamically control the diameter *D*_hex_ of individual skyrmions without modifying the metasurface geometry. Our platform provides a route towards frequency-encoded topological states as reconfigurable building blocks for next-generation quantum photonic platforms.

### Non-local mode formation for the generation of polaritonic skyrmions

The non-local photonic mode that emerges from our structure relies on strong mutual field interactions between individual resonators^34^ that generate out-of-plane electric fields *E*_*z*_ (Fig. 1c). Importantly, these fields are highly uniform across the resonator surface in both amplitude and phase, in contrast to, for example, those emerging from a dipolar resonance (Fig. 1g–i). By covering each resonator with hBN (Fig. 1d) and tailoring the resonance to lie within the in-plane RS band of hBN (Fig. 1e) (where *ε*_*r*,*||*_ < 0), our approach enables in-phase generation of HPhPs^40^ on individual resonators. These modes arise in thin hBN films due to long-range coulomb interactions and the macroscopic polarization field that leads to a spectral splitting between longitudinal and transverse optical phonons, together with the intrinsic anisotropy of hBN. This anisotropy originates from strong in-plane covalent bonding and weaker out-of-plane van der Waals interactions, leading to strong polariton confinement.

A key advantage of our platform is that HPhPs are generated with identical intensity and phase at each resonator edge (Supplementary Fig. 1), unlike traditional approaches using single resonant structures^41–43^. We simulate the out-of-plane electric field *E*_*z*_ of the hBN-covered metasurface and observe constructive interference of HPhPs at the resonator centre, forming a lattice of photonic skyrmions (Fig. 1f). This contrasts with local dipolar resonances in single dielectric resonators (Fig. 1g), which produce non-uniform *E*_*z*_ distributions and polarization-dependent intensity (Fig. 1h and Supplementary Fig. 2). Thus, local resonances such as dipolar modes are fundamentally incapable of generating the relevant topologies (Fig. 1i).

**Fig. 2:**
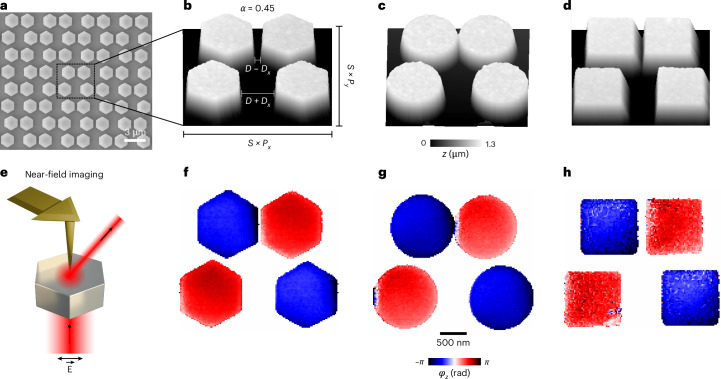
Near-field microscopy of all-dielectric qBICs. **a**, An SEM image of the fabricated metasurface. **b**, An AFM measurement showing the geometry of a single hexagon resonator unit cell with pitches *P*_*x*_ and *P*_*y*_, scaling factor *S* and distances between each resonator pair *D −* *D*_*x*_ and *D* + *D*_*x*_, where *D*_*x*_ determines the radiative loss *γ*_rad._ **c**,**d**, AFM measurements of a single unit cell with discs (**c**) and squares (**d**) as resonators. **e**, A sketch of a metallic s-SNOM tip on top of a dielectric resonator that scatters the local near-field in transmission mode. See the Methods for more information. **f**–**h**, The experimental out-of-plane optical near-field phase *φ*_*z*_ measured on the a-Si metasurface for hexagonal (**f**), disc (**g**) and square (**h**) resonators, showing similar uniform out-of-plane electric field distributions regardless of the resonator shape. The observed phase patterns for all structures agree well with simulations shown in Fig. 1 and Supplementary Fig. 4.

We started our experimental investigation by imaging the all-dielectric metasurface, schematically shown in Fig. 1b. Our design consists of hexagonal amorphous silicon (a-Si) pillars, with each pair laterally offset from one another. A scanning electron microscopy (SEM) image of the fabricated metasurface is shown in Fig. 2a, and atomic force microscopy (AFM) measurements of single unit cells with hexagonal, circular, and square resonators are shown in Fig. 2b–d. To spectrally tune the metasurface resonance, we vary the in-plane scaling factor *S*, which linearly modifies all unit cell dimensions except the height of the a-Si pillars. For all experiments, the pitch was set to *P*_*x*_ = 5,250 nm, *P*_*y*_ = 4,725 nm for a scaling factor *S* = 1 and the height of the resonators to *h*_Si_ = 1,450 nm. For a periodic array of resonators with *C*_4_ symmetry (that is, no lateral offset), the qBIC manifests as a dark mode without radiative loss channels (infinite *Q*-factor) and cannot be observed in the far field. To access this photonic mode experimentally, the in-plane symmetry within each unit cell is broken, opening a radiative loss channel and resulting in an observable resonance (Supplementary Fig. 4) with finite radiative *Q*-factor *Q*_rad_, which can be tuned by offsetting alternating resonator pairs by a distance *D*_*x*_ (Fig. 2b).

To quantify this asymmetry, we define the asymmetry parameter *α* as follows:

1 $$\alpha =\frac{{D}_{{x}}}{{{S \times P}}_{x}}.$$

For all samples fabricated in this work, we choose *α* = 0.045, as it provides relatively high *Q*_rad_ of around 50–100 (Supplementary Fig. 4) while maintaining sufficiently broad resonances to experimentally reconfigure the HPhP wavelength, as the linewidth of the qBIC resonance corresponds to the tuning range of our approach. Such tunability arises from the strong sublinear dispersion of hBN (Supplementary Fig. 4), which enables large changes in polariton wavelength with small changes in excitation frequency^28,42^. To ensure that the uniform out-of-plane electric fields can be accessed over a broad range of HPhP momenta, the metasurface is purposefully designed to support resonances with modest *Q*-factors.

The local near-fields of the photonic qBIC mode are imaged using transmission-mode s-SNOM with a sharp metallic tip (radius ≈ 50 nm) as a local scatterer (Fig. 2e). The full setup is shown in Supplementary Fig. 5. As the tip is polarized along the shaft, it primarily scatters out-of-plane electric fields *E*_*z*_. By focusing a single-wavelength mid-IR beam onto the metasurface at normal incidence and scanning across individual resonators, both the local out-of-plane amplitude |*E*_*z*_| and phase *φ*_*z*_ are extracted via pseudo-heterodyne (PsHet) detection^43^. The measured *φ*_*z*_ for hexagonal resonators is shown in Fig. 2f and agrees well with simulations (Fig. 1c and Supplementary Fig. 4), exhibiting uniform out-of-plane electric fields across each resonator surface. In addition, edge scans on the metasurface (Supplementary Fig. 6) show that the non-local mode forms after approximately 6–7 resonators (3–4 unit cells), indicating that only a few unit cells are required to generate the qBIC mode in the near field, consistent with previous studies^38,39^.

We demonstrate the versatility and generality of our concept by measuring the *φ*_*z*_ of unit cells with modified resonator shapes, namely discs (Fig. 2g) and squares (Fig. 2h), in addition to the hexagonal structures. The results show high uniformity of *E*_*z*_ across all geometries, indicating that the approach is generally applicable to different resonator shapes, provided sufficient mode volume is available for proper formation of the photonic mode. Further details are given in the Methods, and a fabrication sketch is shown in Supplementary Fig. 7. For this study, transmission-mode s-SNOM is preferred over reflection mode, as it enables excitation of the qBIC mode at normal incidence while suppressing tip-launched polaritons^44^. This allows the tip to act as a passive scatterer, detecting near-fields generated by the photonic mode without perturbing the polaritonic topologies.

To generate qBIC-driven photonic skyrmion lattices localized on individual resonators, we fabricated dielectric metasurfaces covered with hBN flakes of thickness *h*_hBN_ = 50–70 nm. A SiO_2_ layer (*h*_SiO2_ = 50 nm) is inserted between a-Si and hBN to enhance adhesion and increase polariton lifetimes owing to its lower refractive index^45^. For all samples, hBN flakes of size 50 × 50 to 100 × 100 μm^2^, covering 10–20 unit cells, were used. To maximize spatial mode density, we employed a spectral gradient metasurface^46,47^ by continuously varying the in-plane scaling factor *S* along one axis, spatially encoding a range of resonance wavelengths within a single array (Supplementary Fig. 8) and reducing the footprint^46^. The spatial encoding was verified via large-area near-field scans (Supplementary Fig. 9).

**Fig. 3:**
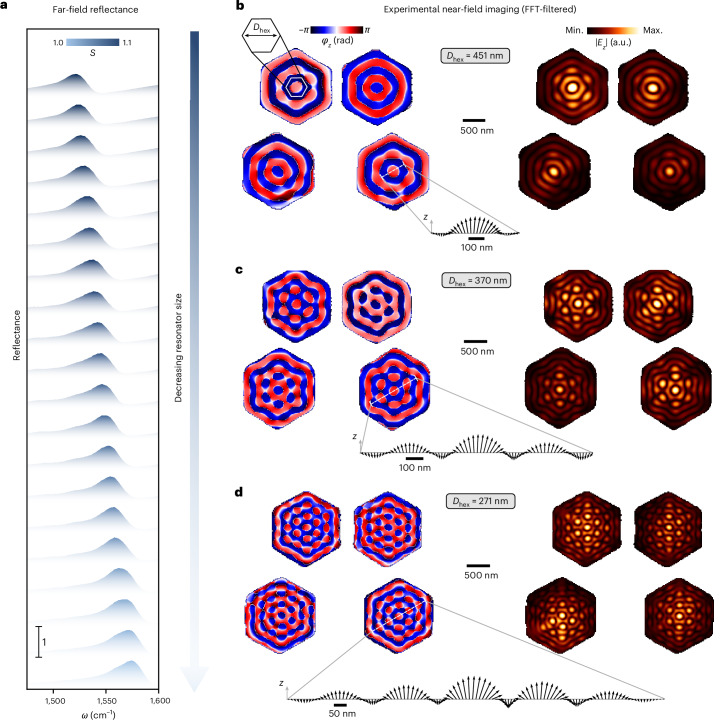
Experimental observation of qBIC-driven photonic skyrmion lattices. **a**, The measured far-field reflectance spectra of fabricated metasurface for varying *S*, exhibiting a shift towards larger wavenumbers when decreasing the unit cell size. **b–d**, Experimental near-field out-of-plane optical phase *φ*_*z*_ (left) and amplitude *|E*_*z*_| (right) images measured in unit cells of *S* varying between 1.1 and 1.0, resulting in HPhP wavelengths of *λ*_HPhP_ *=* 451 nm (**b**), 370 nm (**c**) and 271 nm (**d**). Below each image is a 2D cross section of the electric field vector extracted from the dashed white line marked in the experimental phase images. All measurements were taken on hBN flakes with a thicknesses *h*_hBN_ between 50 and 70 nm and excitation wavenumbers of 1,517 cm^−1^ (**b**), 1,532 cm^−1^ (**c**) and 1,560 cm^−1^ (**d**). Images were filtered using the fast Fourier transform (FFT) procedure described in Supplementary Fig. 12 and Supplementary Note 3, and unfiltered images are shown in Supplementary Fig. 13.

Owing to the high tunability of HPhPs with small shifts in excitation frequency in hBN thin films^42,48^, the broad range of resonances covered by our metasurface (Fig. 3a) generates HPhPs with drastically different wavelengths along the gradient (Fig. 3b–d), resulting in photonic skyrmion diameters ranging from *D*_hex_ = 451 nm down to 271 nm. Note that by definition, *D*_hex_ = *λ*_HPhP_. Individual photonic skyrmions are visible in both the measured phase *φ*_*z*_ and amplitude *|E*_*z*_|, with decreasing size and increasing number per resonator at higher excitation wavenumbers and smaller scaling factor *S*. Our qBIC-driven skyrmions are deeply subwavelength (~*λ*/25) and are about twice as small as plasmonic skyrmions reported previously^17^. This strong confinement arises from the polariton dispersion, which yields large in-plane momenta, leading to an imaginary out-of-plane wavevector and evanescent decay normal to the surface^49^. The HPhP wavelength can be further reduced by increasing the excitation wavenumber or using thinner hBN, potentially combined with sharper tips to resolve smaller features. Note that the volumetric HPhPs, which are typically observed in hBN slabs (>10 nm), would shift towards purely surface modes when approaching the single atomic layer limit^50^. In principle, this enables an arbitrary number of photonic skyrmions on a single resonator, allowing localized and optically reprogrammable topological charges (Supplementary Fig. 10). While hBN supports multiple hyperbolic modes at a given excitation wavelength, the dominant mode (*m* = 0; Supplementary Fig. 3) is primarily detected with s-SNOM^29^. Although we use *α* = 0.045 for all fabricated structures, simulations show that the topology is generated regardless of the resonance *Q*-factor (Supplementary Fig. 11).

### Topological reconfigurability of qBIC-driven polaritonic skyrmions

To characterize the topological properties of qBIC-driven photonic skyrmions, we calculated the skyrmion number density (SND), which describes the spatial distribution of the field’s topological characteristics, and topological winding number *S*_T_, which describes how many times the vector field in a given area *σ* wraps around the unit sphere. These quantities can be written as

2 $$\rm{SND}=\frac{1}{4{\pi }}\hat{\mathbf{e}}\cdot \left(\frac{\partial \hat{\mathbf{e}}}{\partial \it{x}}\times \frac{\partial \hat{\mathbf{e}}}{\partial \it{y}}\right)$$

3 $${S}_{\mathrm{T}}={\int }_{\sigma }\,\rm{SND}\,{\rm{d}}{\it{A}}$$

where $\hat{\mathbf{e}} = (E_{x}, E_{y}, E_{z}) / \sqrt{|E_{x}|^2 + |E_{y}|^2 + |E_{z}|^2}$ is the normalized electric field vector. Hereby, the winding number denotes the number of skyrmions within any given area *σ* on the surface of a resonator. The in-plane electric field components can be directly obtained from the out-of-plane electric field measured with s-SNOM through Maxwell’s equations, as shown in Supplementary Note 4 and in previous works^17,32^.

**Fig. 4:**
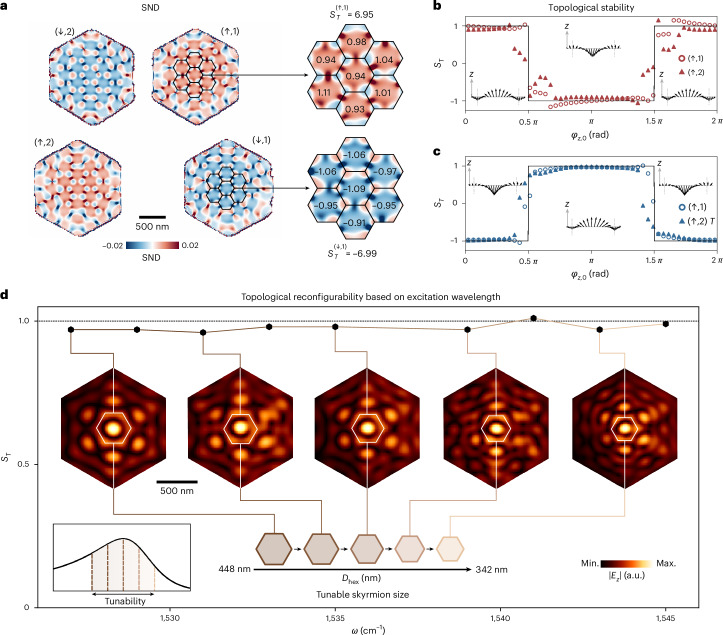
Experimental reconfigurability of qBIC-driven photonic skyrmion lattices. **a**, The SND calculated from the measurements shown in Fig. 3d. Each resonator is marked with a notation showing the out-of-plane electric field direction (↑ or ↓) of the centre skyrmion and a number to distinguish opposing pairs (1 or 2). The resonators (↑, 1) and (↓, 1) are further analysed in the right panels, which show the calculated topological charge within each lattice site being close to the theoretical value of 1. The total topological charges of seven adjacent lattice sites are found to be ${S}_\mathrm{T}^{(\uparrow ,1)}=6.95$ and ${S}_\mathrm{T}^{(\downarrow ,1)}=-6.99$, close to the theoretical values of 7 and −7, respectively. **b**,**c**, The measured topological charge stability of the central lattice site for (↑, 1) (red circles) and (↑, 2) (red triangles) (**b**) and (↓, 1) (blue circles) and (↓, 2) (blue triangles) (**c**), proving the robustness of our photonic skyrmions under continuous tuning of the optical phase *φ*_*z*_. The insets show the respective 2D cross sections through the central lattice site of the measured electric field vector. **d**, The experimental reconfigurability shown by scanning a single resonator repeatedly with different excitation wavenumbers. Inset images show the measured optical amplitude *|E*_*z*_*|* for each excitation frequency. The topological charge *S*_T_ of the central lattice sites consistently stays at the theoretical value of +1 despite sizeable tuning of the skyrmion diameter *D*_hex_. The inset in the lower left shows a sketch of the qBIC resonance and the excitation wavenumbers (dashed brown lines) used for imaging. Unfiltered images are shown in Supplementary Fig. 15.

We study the SND and *S*_T_ for the measurement shown in Fig. 3d (*D*_hex_ = 271 nm). Each resonator is labelled with a notation of (↑, *n*) or (↓, *n*), where ↑ or ↓ denotes the field direction of *E*_*z*_ for the central skyrmion and $n$ distinguishes two resonators of the same polarity. The calculated SND (Fig. 4a) shows a typical Néel-type skyrmion pattern with domain walls that are smeared-out^17^, owing to the deeply subwavelength nature of the HPhPs generated in our structure (*λ*_HPhP_ ≈ *λ*_0_/25). In general, Néel-type photonic skyrmions with smeared domain walls are more readily accessible in materials that support phonon polaritons, as surface plasmon polaritons with long propagation lengths generally exhibit only a moderate reduction in wavelength compared with the incident light^44^. Calculated SNDs for the measurements in Fig. 3b,c are shown in Supplementary Fig. 14.

Within a cluster of seven adjacent hexagonal cells of the skyrmion lattice (each with diameter *D*_hex_), the winding number *S*_T_ is close to the theoretical value of ±1 within each area (Fig. 4a, right), with the sign depending on the direction of *E*_*z*_. Owing to opposing field directions in each resonator pair emerging from the non-local qBIC resonance, both *S*_T_ values of +1 and −1 are recovered within each lattice site. This contrasts with photonic skyrmions in non-resonant isolated structures, where *S*_T_ is solely determined by the phase of the incident light. Summing all winding numbers for the resonators (↑, 1) and (↓, 1) across the seven hexagonal cells yields values of 6.95 and −6.99, respectively, in good agreement with the theoretical value *S*_T_ = ±7, demonstrating the topological robustness of qBIC-driven photonic skyrmion lattices. This stability is further illustrated by smoothly varying the optical phase *φ*_*z*_ (Fig. 4b,c) and calculating the *S*_T_ for each value, where for (↑, 1) (↑, 2) and (↓, 1) (↓, 2), *S*_T_ abruptly switches from +1 to −1 and back to +1, consistent with theoretical modelling.

As illustrated in Fig. 1a, our platform circumvents geometrically wavelength-specific offsets for phase compensation, relying instead on engineered qBIC resonances mediated by long-range coupling between resonators. We demonstrate optical reconfigurability by consecutively imaging the same resonator while tuning the excitation frequency in small steps (∆*ω* = cm^−1^), which changes *λ*_HPhP_ substantially owing to the strong dispersion within the hBN in-plane RS-band. Our measurements (Fig. 4d) show continuous tuning of *D*_hex_ within the same resonator from 448 nm to 342 nm, while the winding numbers *S*_T_ around each central skyrmion remain close to the theoretical value of 1. This tunability window can, in principle, be made arbitrarily large by broadening the qBIC resonance via increasing the asymmetry parameter *α*, enabling the photonic mode to form over a wider wavelength range.

### Generation of arbitrarily structured topologies

Our platform offers a straightforward route to generate arbitrarily structured optical topologies through variation of the resonator shape. As shown in Supplementary Fig. 4, the uniform out-of-plane electric fields of the non-local qBIC metasurface are preserved when moving from hexagonal resonators to discs or squares of comparable mode volume. As with the hexagonal design generating skyrmion lattices, disc and square resonators exhibit the same degree of *E*_*z*_ uniformity across each surface. We expect this behaviour to extend to more complex resonator shapes resembling a disc, such as twisted hexagons for the generation of skyrmion bags.

**Fig. 5:**
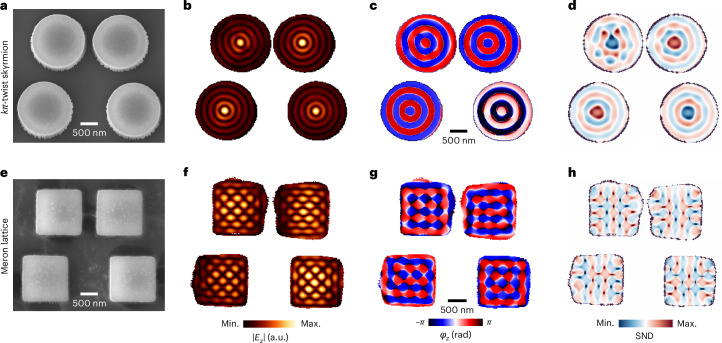
Other qBIC-driven photonic topologies. **a**–**d**, An SEM image (**a**), experimental optical amplitude (|*E*_*z*_|) (**b**), optical phase (*φ*_*z*_) (**c**) and SND (**d**) of an optical *kπ*-twist skyrmion, exhibiting characteristic concentric rings around the centre of the disc. **e**–**h**, An SEM image (**e**), experimental optical amplitude (|*E*_*z*_|) (**f**), optical phase (*φ*_*z*_) (**g**) and SND (**h**) of an optical meron lattice. Simulated and experimentally obtained *S*_T_ values for meron lattices are shown in Supplementary Fig. 17. Unfiltered images are shown in Supplementary Fig. 19.

To demonstrate the generality of our platform, we experimentally probe polaritonic *kπ*-twist skyrmions, previously only observed in singular graphene discs^51^, as well as optical meron lattices, previously realized in patterned gold films^27^ (Supplementary Fig. 16). The measured *kπ*-twist skyrmions are generated in disc resonators (Fig. 5a–d) and consist of concentric rings centred around a single skyrmion with alternating out-of-plane electric field directions. Square resonators instead generate optical meron lattices (Fig. 5e–h), with both simulated and measured *S*_T_ values shown in Supplementary Fig. 17. Note that merons are topologically less stable than skyrmions as they span only half a unit sphere, resulting in *S*_T_ values deviating further from the ideal ±0.5. For large-scale SEM images of the fabricated devices, see Supplementary Fig. 18. As shown previously for the tunable skyrmion lattices in hexagonal resonators, this approach removes the need for wavelength-specific geometries and enables same-structure reconfigurability.

## Conclusion

We report the realization of localized polaritonic topologies arising from a non-local qBIC resonance. By uniting photonic metasurfaces and polaritonic topologies, our approach enables the generation of optically reconfigurable topologies such as skyrmion lattices, meron lattices and *kπ*-twist skyrmions. Our platform moves away from traditional non-resonant singular structures and highly localized resonances. Instead, it leverages non-local photonic modes which rely on long-range resonator-to-resonator interactions to overcome the limitations of conventional designs by enabling optically reconfigurable topologies. As opposed to previous approaches using radially polarized light to generate polaritonic skyrmions^33^, our platform makes use of incident light with spatially uniform polarization, ensuring scalability for future topological computing devices and ease of alignment. Combining the physics of qBIC metasurfaces with topology introduces a fundamentally new tuning knob, enabled by precise control over the modulation and linewidth of the photonic mode, as well as over the phase of the local near fields. Furthermore, the omission of nanoscale offsets and grating periods may enhance the topological robustness of the generated lattices by simplifying fabrication. Our platform may also facilitate the excitation of topological charges through structured topological light fields.

We experimentally realize deeply subwavelength (*λ*/25) optical Néel-type skyrmions with diameters ranging from *D*_hex_ = 45 nm to 271 nm in spectral gradient metasurfaces and confirm their robustness by calculating the topological winding number on each resonator, yielding *S*_T_ = +6.95 and −6.99, close to the expected ±7. We further demonstrate topological reconfigurability on the same structure, tuning the skyrmion diameter from *D*_hex_ = 448 nm to 342 nm without altering its topology. In principle, *S*_T_ can be increased by raising the excitation frequency, allowing each resonator to carry reconfigurable topological charges. Alternatively, thinner flakes or higher-order HPhPs could be used, both leading to stronger wavelength compression. While increased confinement enhances losses, this can be partially mitigated using isotopically enriched hBN, which supports longer propagation lengths^52^. The gradient metasurface framework enables such polaritonic topologies in substantially more compact systems than conventional pixelated approaches^53^, facilitating the use of size-limited van der Waals flakes for probing the interactions between skyrmions and other excited modes at deeply subwavelength scales.

By driving polaritonic topologies via qBIC resonances, our platform could enable the study of near-field phase-engineered topological effects, such as spin in skyrmion lattices or orbital angular momentum in polaritonic vortices. The strong field confinement and deeply subwavelength nature of these skyrmions are promising for information encoding, photonic computing and high-precision metrology^54^. Combining qBIC metasurfaces with photonic skyrmions may enable exotic light–matter states, including nonlinear skyrmions and skyrmion–skyrmion interactions, with polaritonic materials enhancing nonlinear effects^55^. Metasurfaces have also enabled lasing^56,57^, suggesting the possibility of skyrmion lasing, previously predicted only in microring cavities^58^. Skyrmion and related lattices, such as Kagome lattices, can further be used for particle trapping, sorting and detection^32^, including microplastics. Tunable metasurfaces incorporating phase-change materials^59^ could extend spectral tunability, while the compact footprint of qBIC modes allows substantial miniaturization. In practice, arrays as small as 7 × 7 unit cells (35 × 35 μm^2^) are sufficient to generate robust skyrmion lattices^38,39^.

Finally, we experimentally demonstrate qBIC-driven optical *kπ*-twist skyrmions and meron lattices by modifying the resonator shape while preserving the non-local photonic mode. These topologies can be tuned analogously to the skyrmion lattices shown in Fig. 4. The photonic mode remains robust against variations in resonator shape, enabling combining multiple geometries within a single metasurface to generate quasi-arbitrary structured topologies (Supplementary Fig. 20). More complex geometries, such as dodecagons with alternating angles, could enable reconfigurable skyrmion bags^24^ and broader classes of optical topologies, paving the way for tunable photonic twistronics in a single deeply subwavelength resonator. Similarly, pentagonal metasurfaces could realize tunable topological quasi-crystals for exploring four-dimensional topological charge vectors^60^. Overall, our approach brings polaritonic skyrmions to the integrated photonic chip scale, enabling multiplexing through spatial encoding of individual topologies.

## Methods

### Numerical simulations

All numerical simulations shown in this work were conducted using CST Studio Suite (Simulia), a commercial finite element solver. The setup includes adaptive mesh refinement and periodic boundary conditions in the *x*,*y* direction and open boundaries in the *z* direction. All simulations were conducted in the frequency domain. The numerically calculated near-fields were extracted 10 nm above the surface of the hBN layer to replicate the experimental s-SNOM measurements. The electric fields were then exported with 5-nm resolution to capture the highly fluctuating field patterns present in optical skyrmion lattices. The permittivities of hBN and SiO_2_ were taken from ref. ^42^ and ref. ^61^, respectively. The permittivities of Si and CaF_2_ were set to 10.6 and 2.05, respectively.

### Optical near-field measurements

All near-field experiments were conducted with a commercial s-SNOM system (neaSCOPE, Attocube Systems). The laser source was a continuous wave QCL (MirCat, Daylight Solutions), tunable from 1,755 cm^−1^ to 1,315 cm^−1^, which covers the entire range of the hBN RS band. All measurements were done in transmission mode in order to ensure normal incidence to properly excite the qBIC mode. To study the topological properties of qBIC-driven optical skyrmions, both near-field scattering amplitude *|E*_*z*_*|* and phase *φ*_*z*_ need to be obtained, which required the use of PsHet^43^. To accomplish this, the beam is first split in two by passing through a beam splitter. One part is loosely focused onto the metasurface and the probing AFM tip at 0° incidence from below (through the CaF_2_ substrate) via a parabolic mirror. The tip is operated in tapping mode, oscillating at a frequency of around 250 kHz. For all experiments, we used metal-coated (Pt/Ir) AFM tips (Arrow-NCPt, NanoWorld). The light backscattered from the tip is then re-collected through a second parabolic mirror. The second part of the beam passes through a delay stage, in which two mirrors vibrate with a defined mirror amplitude to achieve decoupling of optical amplitude and phase. When conducting PsHet interferometry^62^, the frequency of vibration for these two mirrors (around 300 Hz) is typically much lower than the tip frequency. Both beams are recombined at a second beam splitter and measured with a liquid nitrogen cooled MCT-detector. To distinguish the near-fields from far-field background scattering, the signal is demodulated at higher tip harmonics (*n* > 2 for all measurements), ensuring that only the local near-fields generated on top of each resonator are detected. In addition, PsHet enables the elimination of the multiplicative background contribution otherwise present in all s-SNOM experiments. The reference mirror introduces side peaks next to the main tip harmonics, which do not contain the multiplicative background, thus demodulation at the side band frequencies of sufficiently high *n* allows for conducting near-field measurements without any far-field contributions^43^.

### Fabrication

To fabricate the skyrmion qBIC gradient structures, the process began with the deposition of an amorphous silicon layer via plasma-enhanced chemical vapour deposition using a PlasmaPro 100 system (Oxford Instruments). This was followed by the application of a conformal SiO_2_ layer through radio-frequency sputtering on an Amod PVD system (Angstrom Engineering). High-quality hBN flakes were then mechanically exfoliated from bulk crystals (HQ Graphene) and transferred onto the substrate, which was maintained at 160 °C to eliminate residual moisture and promote clean adhesion by softening the adhesive tape. Any remaining tape residues were removed using a 10-min oxygen plasma treatment. Flakes with adequate lateral dimensions and uniform thickness were preselected via optical microscopy and subsequently characterized using a Bruker Dektak XT profilometer to verify their height. Next, a chromium layer was sputtered using the same PVD tool to complete the material stack. The surface was spin-coated with the positive-tone electron beam resist CSAR 62 (AR-P 6200.13, Allresist), and nanoscale patterning was carried out using a Raith eLINE Plus electron beam lithography system operated at 20 kV with a 15-μm aperture. Development proceeded in two steps: an initial bath in amyl acetate, followed by a second stage in a 1:9 mixture of methyl isobutyl ketone and isopropanol. Layer-by-layer pattern transfer was achieved via reactive ion etching on the PlasmaPro 100 system, proceeding in the sequence: chromium (serving as a hard mask after resist removal), hBN, SiO_2_, silicon and a final chromium etch to eliminate the remaining mask. A schematic overview of the fabrication steps is shown in Supplementary Fig. 7.

## Online content

Any methods, additional references, Nature Portfolio reporting summaries, source data, extended data, supplementary information, acknowledgements, peer review information; details of author contributions and competing interests; and statements of data and code availability are available at 10.1038/s41565-026-02174-5.

## Supplementary information

Supplementary Information Supplementary Notes 1–4 and Figs. 1–20.

Peer Review File

Supplementary Video 1 Evolution of experimentally measured skyrmion lattices shown in Fig. 3d, obtained by adding a constant optical phase offset *φ*_*z*,0_ to the measured data.

## Data Availability

The data that support the findings of this study are available via Zenodo at 10.5281/zenodo.19206801 (ref. ^63^).
