# Supplementary material for: Tunable polaritonic topologies generated by non-local photonic modes
Source: Nat Nanotechnol. 2026 May 12;21(6):794–802. doi: 10.1038/s41565-026-02174-5 (PMC13293870; doi:10.1038/s41565-026-02174-5)
Supplement: Supplementary file 1 — Supplementary Notes 1–4 and Figs. 1–20. [file 41565_2026_2174_MOESM1_ESM.pdf]

nature nanotechnology

Supplementary information

https://doi.org/10.1038/s41565-026-02174-5

Tunable polaritonic topologies generated by non-local photonic modes

In the format provided by the authors and unedited

Contents

Supplementary Notes 2
Supplementary Note 1: Dielectric function of hBN 2
Supplementary Note 2: Dispersion of HPhPs in hBN thin films 2
Supplementary Note 3: Data Processing 3
Supplementary Note 4: Calculating the in-plane electric field components 3
Supplementary Videos 4
Supplementary Video 1: Measured qBIC-driven skyrmion lattices 4
Supplementary Figures 4
Supplementary References 14

Supplementary Notes

Supplementary Note 1: Dielectric function of hBN

The in-plane complex permittivity $\varepsilon_{||}$ of hBN (**Fig. 1e**) can be modelled as a Lorentz oscillator of the form:

$$\varepsilon_{||}(\omega) = \varepsilon_{||,\infty}(1 + \frac{\omega_{LO,||}^2 - \omega_{TO,||}^2}{\omega_{TO,||}^2 - \omega^2 - i\omega\gamma_{||}}) \quad (1)$$

where $\varepsilon_{||,\infty}$ denotes the permittivity at high frequencies, $\omega_{TO,||}$ and $\omega_{LO,||}$ are the frequencies of the in-plane transverse and longitudinal optical phonon respectively and $\gamma_{||}$ the damping constant. For all simulations and calculations conducted in this work, $\varepsilon_{||,\infty} = 4.9$, $\omega_{LO,||}^2 = 1614$ cm^{-1}, $\omega_{TO,||}^2 = 1360$ cm^{-1}, and $\gamma_{||} = 7$ cm^{-1}. The values were taken from Ref.[1].

Supplementary Note 2: Dispersion of HPhPs in hBN thin films

To analytically describe the in-plane dispersion of HPhPs in hBN, one can solve Maxwell's equations and find the in-plane momentum corresponding to an incident wave of light at 0° angle of incidence. The derivation can be found in Ref.[2]. We consider a thin slab of hBN on a substrate with a permittivity ε_{sub}. For large enough in-plane momenta, we can approximate the out-of-plane momentum as $k_z = \sqrt{k_0^2 - k_{||}^2} \approx ik_{||}$, resulting in the following analytical expression for k_z:

$$k_z = -\frac{1}{h_{hBN}} \frac{\sqrt{\varepsilon_{||}}}{i\sqrt{\varepsilon_{\perp}}} (-\pi m + \tan^{-1}\frac{i}{\sqrt{\varepsilon_{\perp}}\sqrt{\varepsilon_{||}}} + \tan^{-1}\frac{i\varepsilon_{sub}}{\sqrt{\varepsilon_{\perp}}\sqrt{\varepsilon_{||}}}) \quad (2)$$

where $\varepsilon_{||}$ and $\varepsilon_{\perp}$ are the in- and out-of-plane permittivities respectively and h_{hBN} the thickness of the hBN layer. As is well known from previous works[2], hBN supports multiple modes with different k_z for each frequency inside the Reststrahlen band, denoted by the index m. However, in the case of using silicon as substrate, the dominant mode that is observed and yields the highest near-field scattering intensity corresponds to $m = 0$. However, if the higher order modes ($m > 0$) could be reliably observed through near-field optical microscopy, this would open up the possibility of generating multiple topologies on the same resonator that are even more deeply subwabvelength, since $m > 0$ results in larger momenta.

To simplify, we assume that the substrate consists of silicon with $\varepsilon_{sub} = 10.6$ (realistic for amorphous silicon) and calculate the resulting dispersion (**Fig. S3**) for various thicknesses, as well as the reflection coefficient for different incident frequencies and $k_{||}$. We observe an increase in in-plane momenta for thinner flakes, as well as multiple modes appearing, with $m = 0$ being the most dominant. However, to accurately model the platform used in our experiments and calculate the theoretical HPhP wavelength for our system, both the permittivities of the silicon substrate and the thin layer of SiO_2 ($h_{SiO2} = 50$ nm) present inside the structure should be considered. On top of being helpful in ensuring proper adhesion between the dielectric structures and the hBN flakes, the SiO_2 layer reduces the losses of the

propagating HPhPs due to its lower permittivity. To ensure proper modeling, one could use effective medium theory or transfer matrix methods to estimate an effective permittivity that represents the contributions from both materials.

Supplementary Note 3: Data Processing

To properly evaluate the topological properties of qBIC-driven topologies, it is crucial to filter out high frequency components, as well as properly separating near-field amplitude $|E_z|$ and phase $\varphi(E_z)$. To accomplish this, we first consider the experimentally measured amplitude and phase images and calculate the out-of-plane complex electric field $E_z = |E_z|e^{i\varphi(E_z)}$. We threshold $E_z$with the simultaneously measured topography (threshold at 90-95%) in order to remove the background scattering measured on the substrate and only consider the near-field measured at the top of the resonator (**Fig. S12a**). The images are then separated, with each resonator being analyzed individually (**Fig. S12b**). We apply zero padding to artificially increase the number of pixels (number of pixels is increased by 500%) and multiply the complex electric field with a Gaussian Filter to reduce the influence of the sharp resonator edges on the resulting Fast Fourier Transform (FFT). The FFTs for each resonator (**Fig. S12c**) are filtered (**Fig. S12d**) by selecting a region around the observed HPhP wavelength (±5-10%) and removing both low and high frequency components that do not contribute to the topological fields. The filtered near-field amplitude and phase images can be obtained through inverse FFT (**Fig. S12e**) and are then used to calculate the topological charges shown in **Fig. 4**.

Supplementary Note 4: Calculating the in-plane electric field components

In s-SNOM, the tip mainly scatters the out-of-plane electric field due to the tip shaft being polarized in z-direction. Since optical skyrmions are 2D vector fields, the in-plane components of the electric field need to be known in order to characterize the topologies and calculate the skyrmion number S_T (eq. (3) in the main text). As has been shown rigorously in previous works[3], the in-plane electric field components E_x and E_y of a confined surface wave can be obtained straightforwardly with the following relations:

$$\begin{pmatrix} E_x \\ E_y \end{pmatrix} = \frac{1}{k_{||}^2}\begin{pmatrix} \frac{\partial E_z}{\partial x \partial z} \\ \frac{d^2 E_z}{\partial y \partial z} \end{pmatrix} = \frac{-k_z}{k_{||}^2}\begin{pmatrix} \frac{\partial E_z}{\partial x} \\ \frac{\partial E_z}{\partial y} \end{pmatrix} \tag{3}$$

where k_z and $k_{||}$ are the out-of-plane and in-plane momenta respectively. Hereby, we can write as $k_z = \sqrt{k_0^2 - k_{||}^2} \approx ik_{||}$ for sufficiently large in-plane momenta, since the wavelengths of measured HPhPs in hBN are usually on the order of $\lambda/10 - \lambda/50$. Since s-SNOM yields a 2D map of the sample surface, one can easily obtain the in-plane fields by taking the derivative of E_z along x- and y-direction respectively. Using eq. (3), the electric field vectors of individual skyrmions (**Figs. 3** and **4**) can be fully reconstructed from the measured E_z.

Supplementary Videos

Supplementary Video 1: Measured qBIC-driven skyrmion lattices

Evolution of experimentally measured skyrmion lattices shown in **Fig. 3d**, obtained by adding a constant optical phase offset $\varphi_{z,0}$ to the measured data.

Supplementary Figures

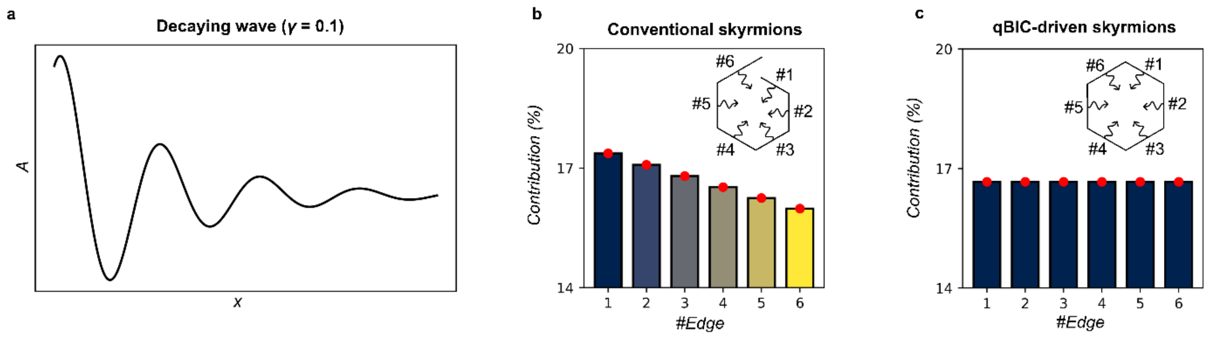

Figure S1. Edge-dependent intensity contribution of phonon-polariton to photonic skyrmion lattice. a Amplitude vs. position of a decaying wave of the form $A = A_0 e^{-\gamma x} cos\,(2\pi \frac{x}{\lambda} + \varphi)$, where A_0 is an amplitude and $\gamma = 0.1$. Analytically calculated contribution to the intensity of the central skyrmion of propagating phonon polaritons in the case of (**b**) the conventional approach using structural offsets for phase correction and (**c**) our nonlocal resonance-driven approach. The inset shows the structures used to generate the skyrmion lattices in each case.

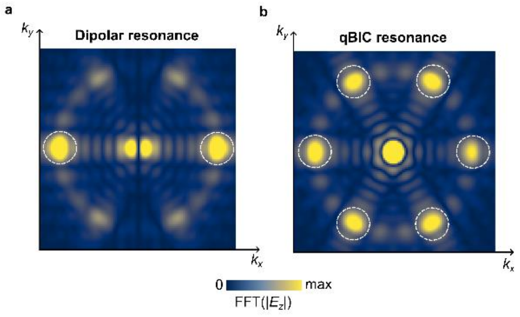

Figure S2. Simulated HPhPs in momentum space of dipolar resonance vs. qBIC-driven skyrmion lattice. a, b Absolute value of the Fourier-transformed out-of-plane electric field E_z in the case of a local dipolar resonance (a) and nonlocal qBIC resonance (b). The white dashed circles show the region of momenta where HPhPs are propagating at high intensity.

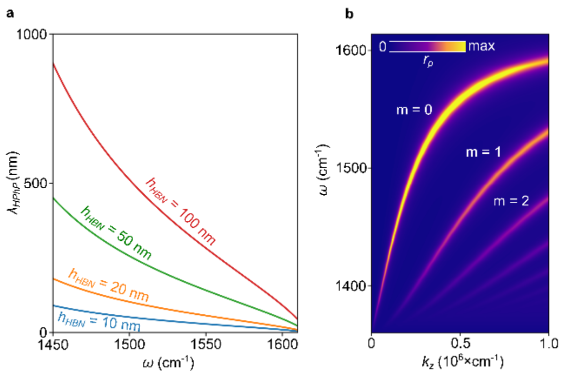

Figure S3. Calculated hBN dispersion. a Calculated polariton wavelength λ_{HPhP} vs. incident frequency ω for different hBN flake thicknesses (see **Supplementary Note 3**, eq. (2)). **b** Reflection coefficient for various ω and $k_{||}$, showing the various hyperbolic polariton branches (m = 0, 1, 2, …).

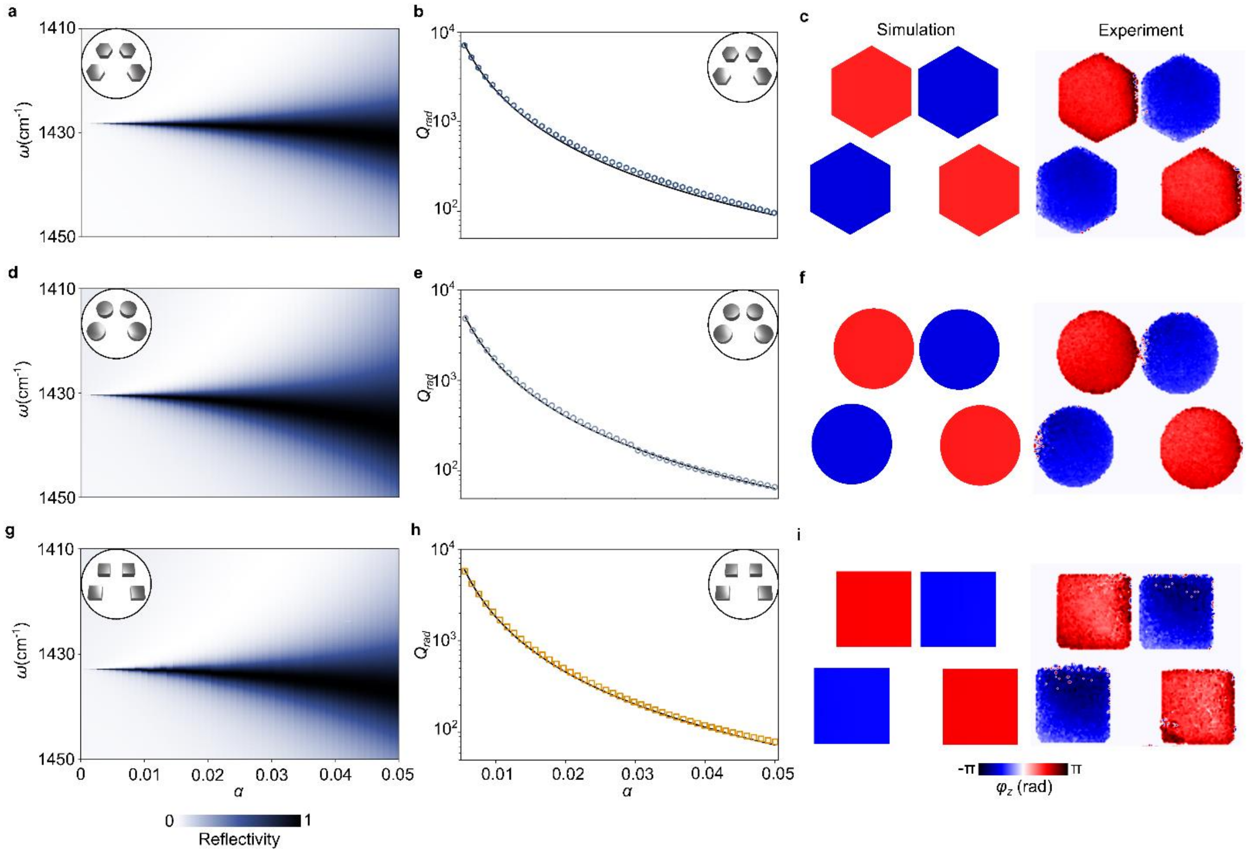

Figure S4. All-dielectric qBIC resonances for various resonator shapes. a Simulated reflectivity for different excitation wavenumbers ω and asymmetry parameters α for a hexagonal resonator metasurface. The resonance broadens as α increases. **b** Radiative Q-factor Q_{rad} vs. α extracted from a Fano lineshape fit to **a**. Full curve shows a fit for $Q_{rad} = c\alpha^{-2}$. **c** Simulated (left) and measured (right) phase of the out-of-plane electric field φ_z of the hexagonal resonator metasurface, displaying a uniform field distribution on the surface of each resonator. **d-f** Same as a-c but using disks as resonators. **g-i** Same as **a-c** but using squares as resonators. The insets show the unit cell used for the simulation in each case.

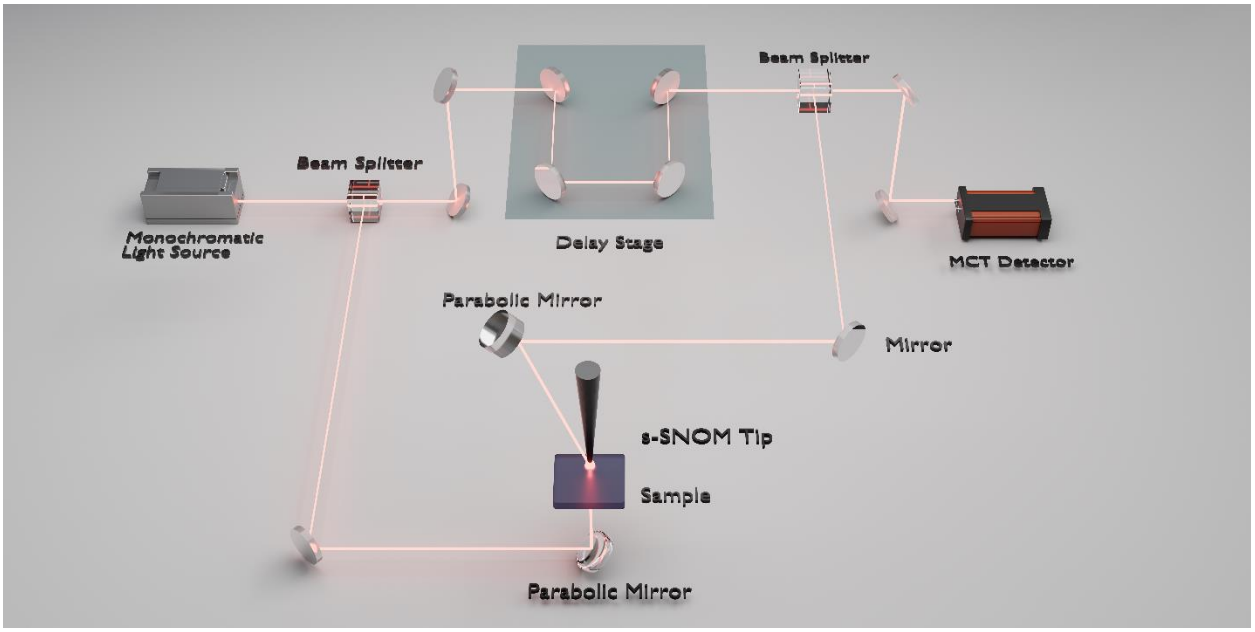

Figure S5. Setup for transmission-mode phase-resolved near-field imaging. A light source generates a continuous wave, monochromatic beam that is split in two at a beamsplitter, with one part acting as a reference beam travelling through a delay stage, and the other travelling to the tip and sample (signal beam), in a Mach-Zehnder configuration. The signal beam is first loosely focused onto the sample with a parabolic mirror and illuminates a sharp metallic tip (r = 50 nm), creating a spatially confined optical hot spot at the tip apex which locally excites the fabricated resonant structures. The tip scatters the resulting out-of-plane near-field back into the far-field, which is collected by a second parabolic mirror. The tip vibrates at a frequency of $\Omega \approx 250$ kHz and the signal is demodulated at harmonics $n\Omega$ to suppress unwanted background ($n > 2$ for all measurements). The signal beam is then overlapped with the reference beam at a second beamsplitter and the interference is measured with a liquid nitrogen-cooled mercury cadmium telluride (MCT) detector. Through pseudo-heterodyne detection using two vibrating mirrors inside the Mach-Zehnder interferometer, both amplitude $|E_z|$ and phase φ_z of the out-of-plane electric field are obtained.

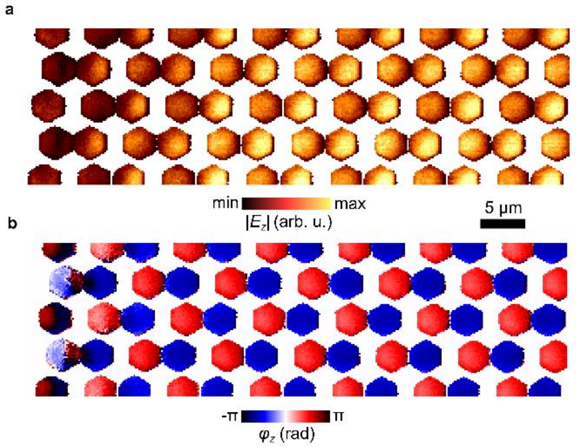

Figure S6. Edge effects of a hexagonal resonator metasurface. a Out-of-plane near-field amplitude $|E_z|$ and **b** phase φ_z of the left edge of an all-dielectric hexagonal resonator metasurface shown in Fig. 2.

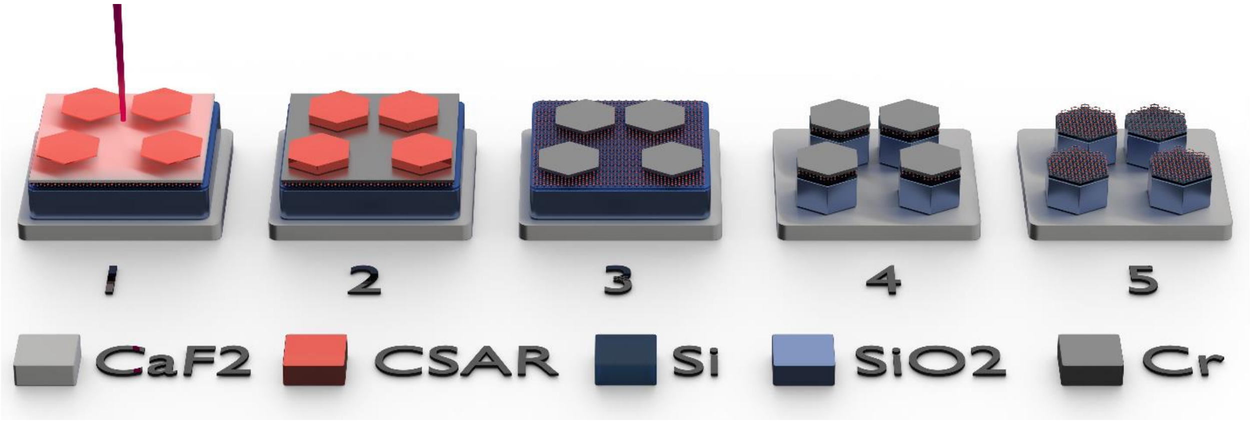

Figure S7. Fabrication process for the skyrmion qBIC gradient metasurfaces. (1) The process begins with spin-coating of CSAR resist (t = 400 nm) onto the multilayer stack (CaF_2 (h_{CaF2} = 1 mm)/Si (h_{Si} = 1.45 μm)/SiO_2 (h_{SiO2} = 50 nm)/hBN (h_{hBN} = 50-100 nm)/Cr (h_{Cr} = 100 nm)), followed by electron beam lithography to define the negative of the desired hexagonal structures. (2) After development, the unexposed resist remains and serves as a hard mask for (3) pattern transfer of the into the underlying chromium layer. (4) These chromium patterns act as a robust hard mask for the subsequent reactive ion etching steps that transfer the structures into the underlying hBN, SiO_2, and Si layers, yielding the (5) final structure.

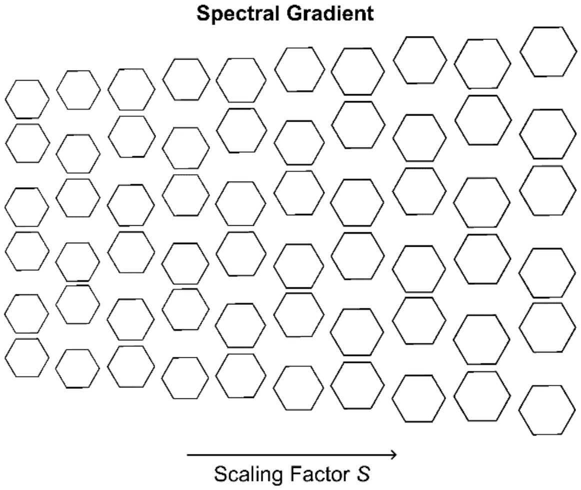

Figure S8. Sketch of a gradient metasurface consisting of hexagonal resonators. The scaling factor is varied along one axis of the metasurface, allowing for smooth spatial encoding of the resonant wavelength across the array.

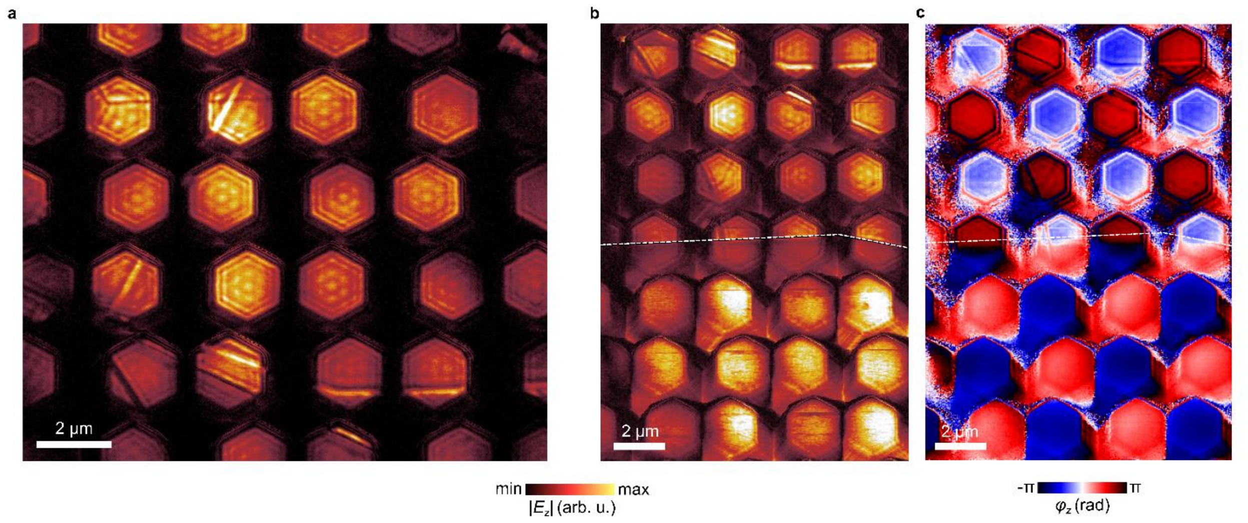

Figure S9. Large-scale near-field imaging of photonic skyrmion lattices. a Out-of-plane near-field amplitude $|E_z|$ of multiple resonators supporting photonic skyrmion lattices. **b, c** Out-of-plane near-field amplitude $|E_z|$ and phase φ_z of multiple resonators, showing the transition between hBN covered and all-dielectric metasurface. White dashed line shows the edge of the hBN flake covering parts of the metasurface.

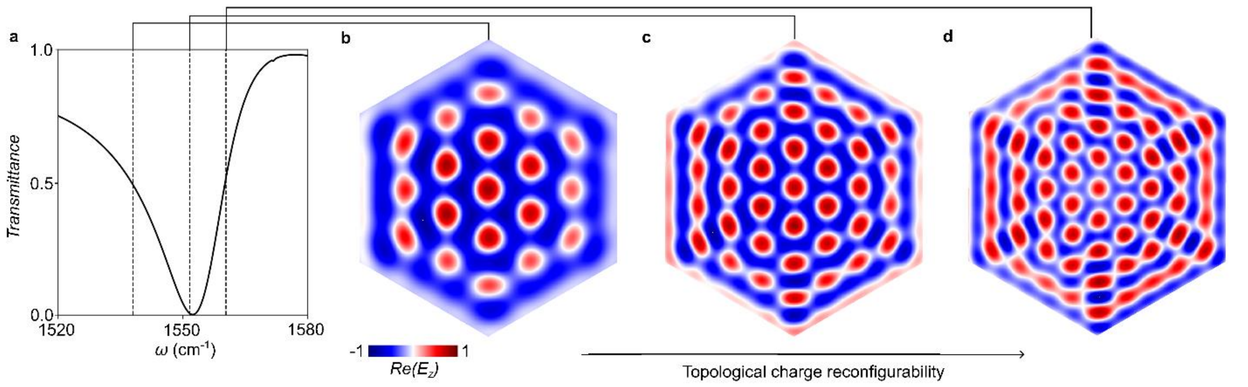

Figure S10. Simulations for reconfigurable topological charges. a Simulated transmittance of a qBIC resonance for $S = 1.08$ and $\alpha = 0.045$. Dashed black lines show the excitation wavenumbers at which the real part of the out-of-plane electric field $Re(E_z)$ is simulated in **b**-**d**. Depending on the excitation frequency ω, a varying number of photonic skyrmions are observed within the same structure through small changes in excitation wavelength.

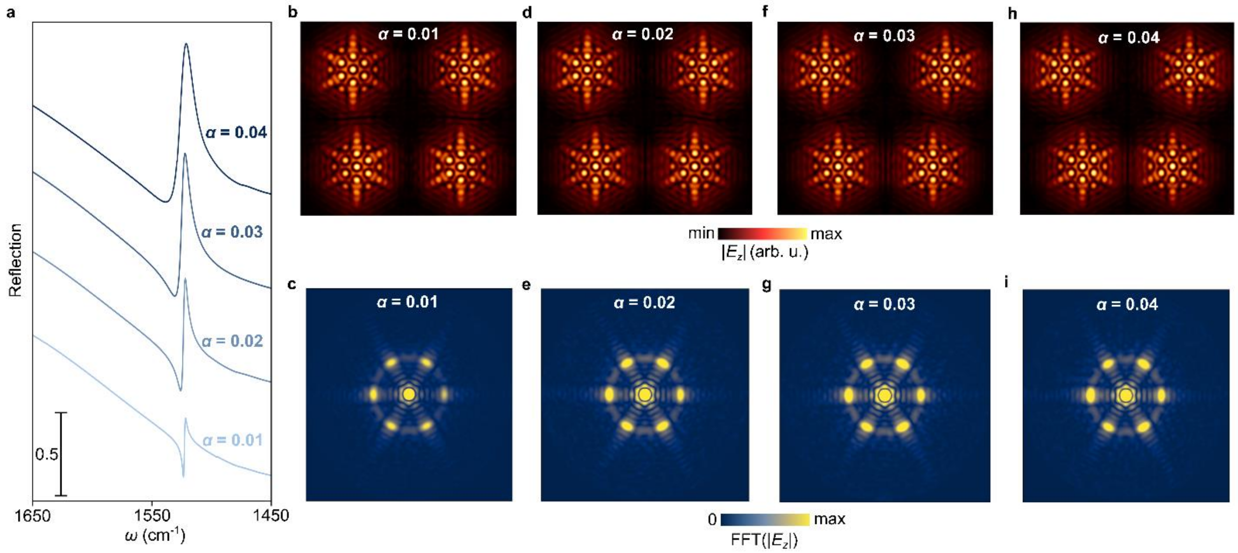

Figure S11. Polaritonic skyrmion lattices for different *Q*-factors. a Simulated reflectance spectra vs. asymmetry parameter *α*. Fourier-filtered out-of-plane near-field amplitude |E_z| and absolute value of the Fourier-transformed out-of-plane electric field E_z for *α* = 0.01 (**b**, **c**), *α* = 0.02 (**d, e**), *α* = 0.03 (**f, g**) *and α* = 0.04 (**h**, **i**). All experiments were conducted on structures with *α* = 0.045. *Q*-factors shown are between 50-200.

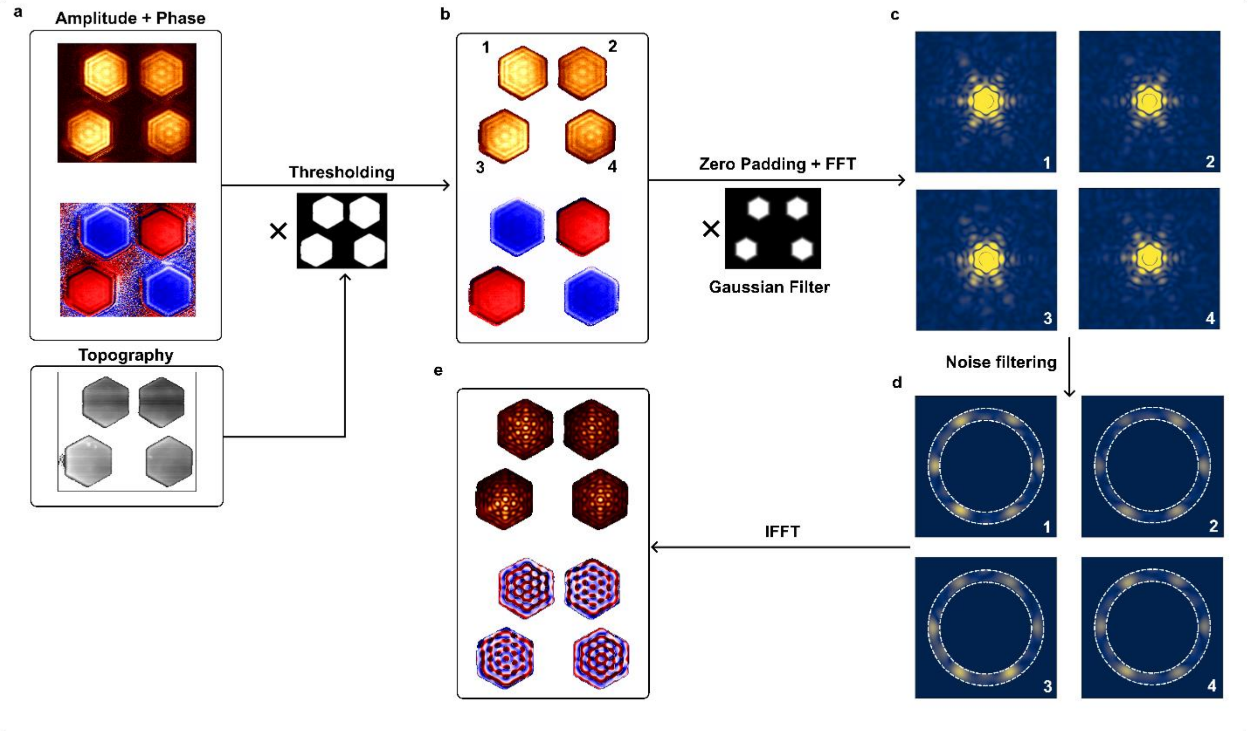

Figure S12. Workflow of data analysis. A threshold is applied to the near-field amplitude and phase (**a**) obtained from s-SNOM measurements (**b**) using the simultaneously acquired topography to isolate each structure. Hereby, 0.9-0.95 of the maximum value measured in the topography is taken as a threshold value. To enhance the resolution, the image is zero-padded and then Fourier-transformed. To avoid sudden jumps from the previous thresholding that could create artefacts in the Fourier-transformed image, we apply a Gaussian filter to smooth the edges. The resulting

Fourier-transformed image (**c**) is then filtered for each resonator individually by choosing a circular region around the polariton wavelength (**d**) (white dashed circles). The image is then transformed back into real space, and amplitude and phase are extracted for further analysis (**e**).

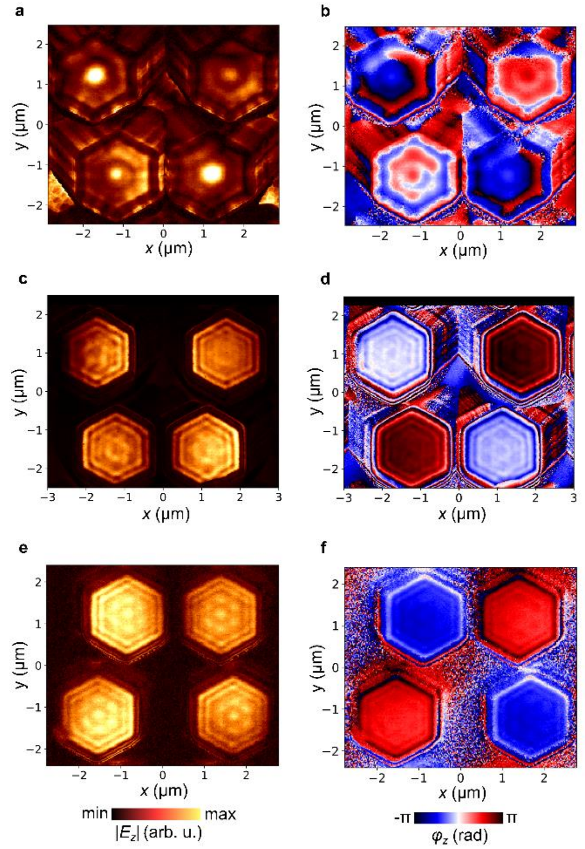

Figure S13. Unfiltered near-field images corresponding to measurements shown in Fig. 3. Excitation wavenumbers used were 1517 cm^{-1} (**a, b**), 1532 cm^{-1} (**c, d**) and 1560 cm^{-1} (**e, f**).

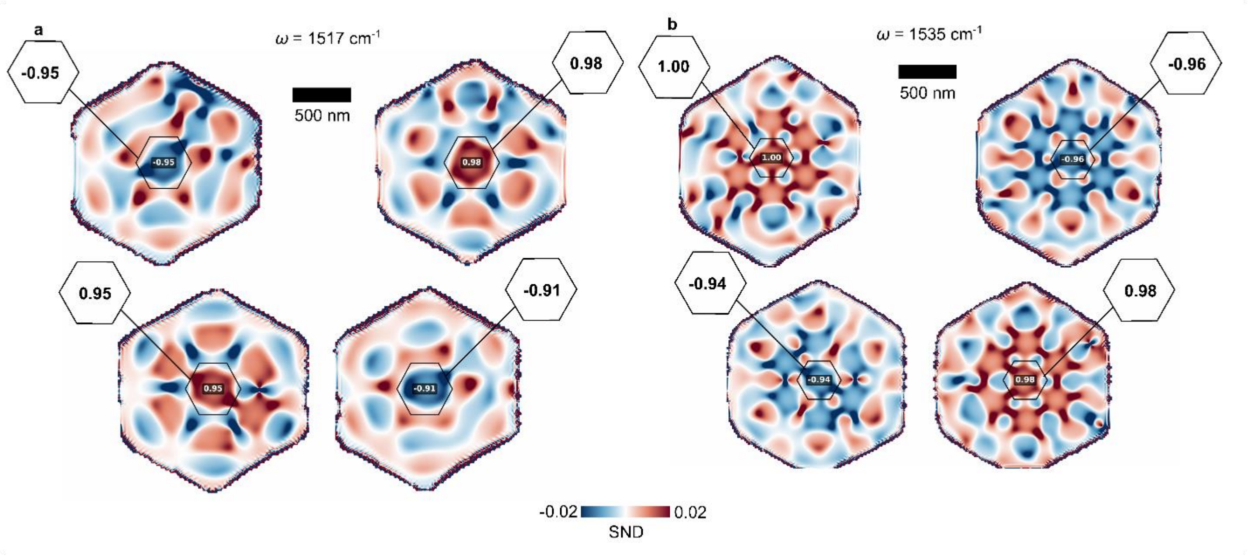

Figure S14. Calculated skyrmion number density *SND* of images shown in Fig. 3b, c. Excitation wavenumbers used were 1517 cm^{-1} (**a**) and 1532 cm^{-1} (**b**). Scale bar 500 nm.

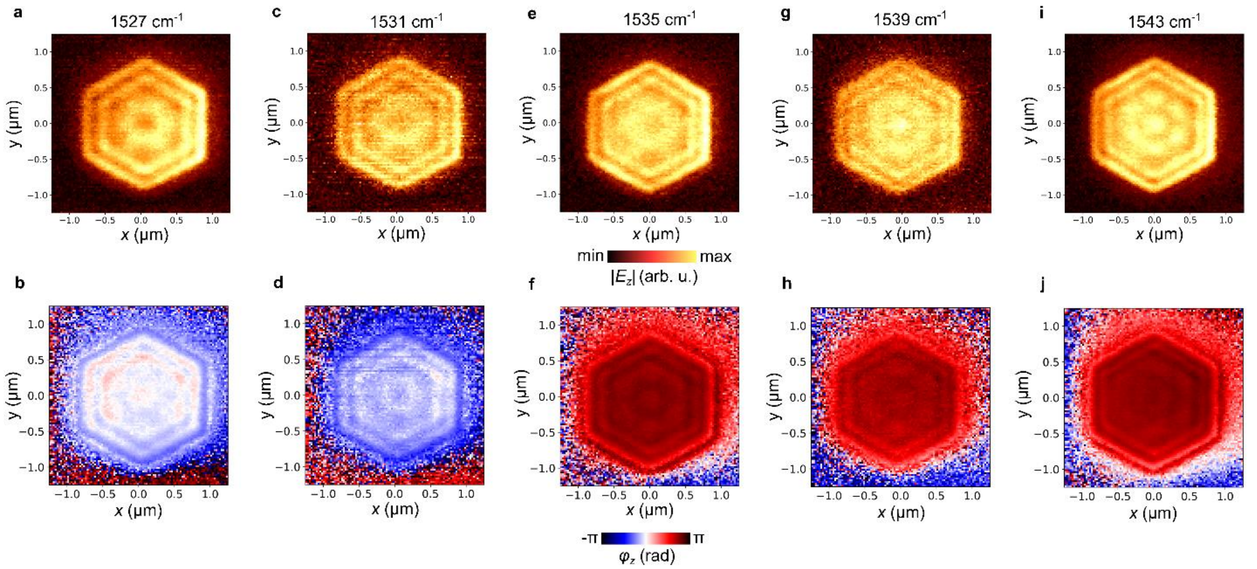

Figure S15. Unfiltered near-field images corresponding to measurements shown in Fig. 4d. Excitation wavenumbers used were 1527 cm^{-1} (**a, b**), 1531 cm^{-1} (**c, d**), 1535 cm^{-1} (**e, f**), 1539 cm^{-1} (**g, h**) and 1543 cm^{-1} (**i, j**).

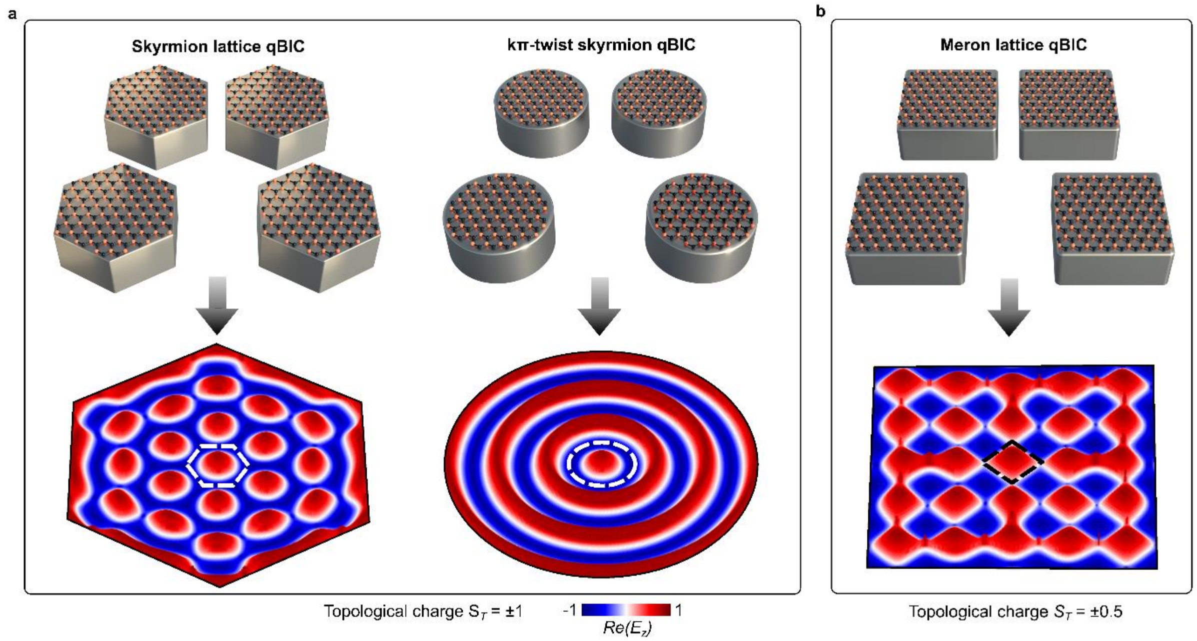

Figure S16. Generation of various qBIC-driven optical topologies. a Hexagonal (left) and disk (right) resonator metasurface generating skyrmion lattices and $k\pi$-twist skyrmions respectively. The topological charge within each cell (white dashed line) is $S_T = \pm1$. **b** Square-shaped resonator metasurface generating meron lattices. The topological charge within each cell (white dashed line) is $S_T = \pm0.5$. Out-of-plane electric field plots were calculated analytically by interfering plane waves, with the boundaries determined via their respective geometries.

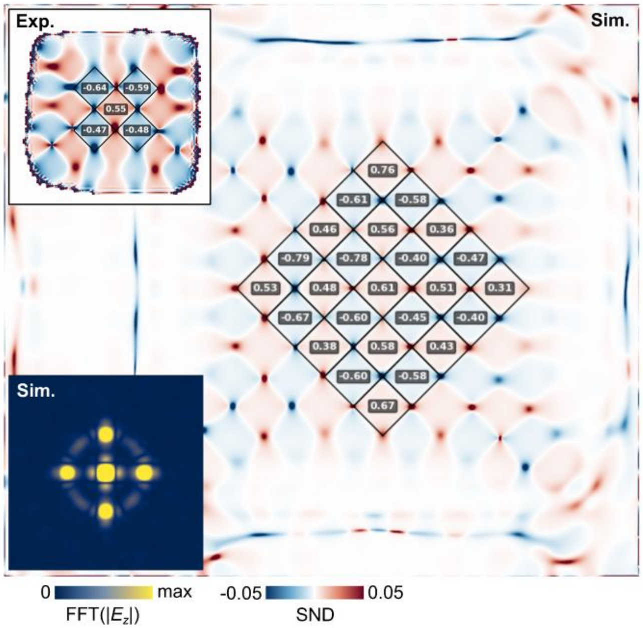

Figure S17. Stability of qBIC-driven merons. Simulated SND and FFT of $|E_z|$ and experimentally measured SND (from **Fig. 5**), with calculated S_T written within each lattice site.

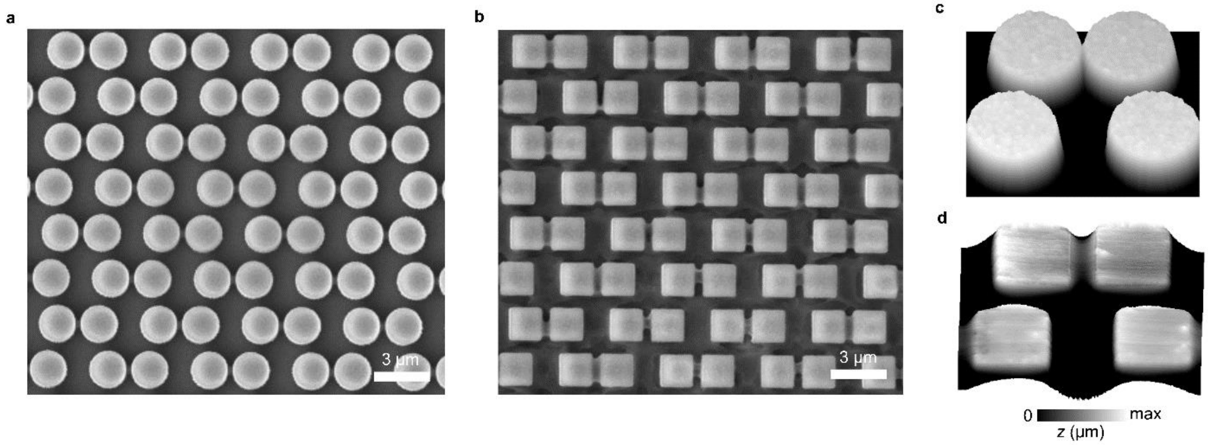

Figure S18. SEM and AFM images of *kπ*-twist skyrmions and meron lattice generating metasurfaces. **a, b** SEM images showing an array of disks and squares that support a qBIC resonance used to generate *kπ*-twist skyrmion and meron lattices respectively. **c, d** AFM image of a single unit cell of the metasurfaces shown in **a, b**.

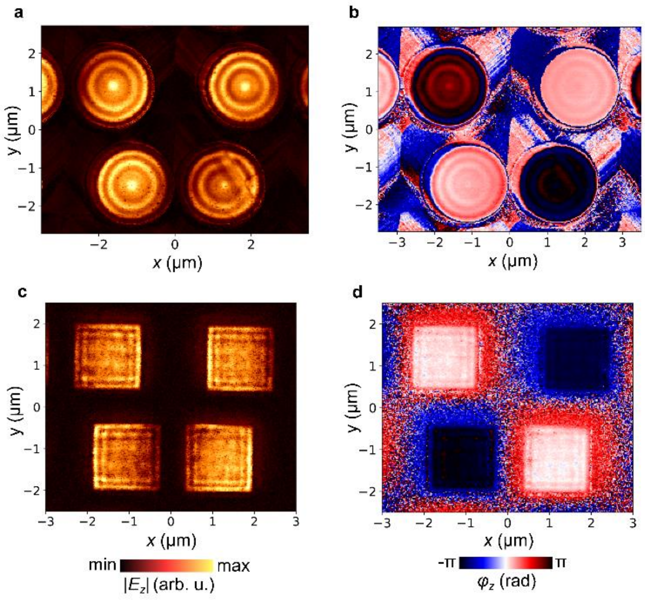

Figure S19. Unfiltered near-field images corresponding to measurements shown in Fig. 5. Excitation wavenumbers used were 1513 cm^{-1} (**a, b**) and 1555 cm^{-1} (**c, d**).

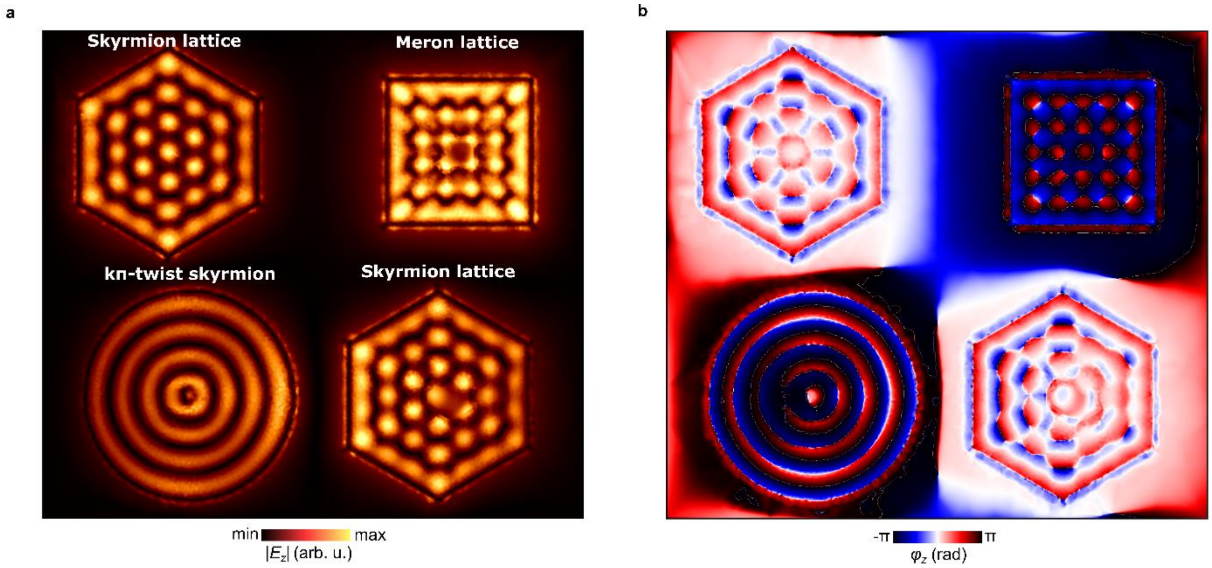

Figure S20. Simulations of a multiplexed unit cell. a Simulated out-of-plane near-field amplitude $|E_z|$ and **b** phase φ_z of a periodic array consisting of a unit cell with two hexagonal resonators (generates skyrmion lattice), a square resonator (generates meron lattice) and a disk (generates kπ-twist skyrmion).

Supplementary References

1. Caldwell, J. D. *et al.* Sub-diffractional volume-confined polaritons in the natural hyperbolic material hexagonal boron nitride. *Nature communications* **5,** 5221; 10.1038/ncomms6221 (2014).

2. Dai, S. *et al.* Tunable phonon polaritons in atomically thin van der Waals crystals of boron nitride. *Science (New York, N.Y.)* **343,** 1125–1129; 10.1126/science.1246833 (2014).

3. Tsesses, S. *et al.* Optical skyrmion lattice in evanescent electromagnetic fields. *Science (New York, N.Y.)* **361,** 993–996; 10.1126/science.aau0227 (2018).
