# Supplementary material for: Tunable polaritonic topologies generated by non-local photonic modes
Source: Nat Nanotechnol. 2026 May 12;21(6):794–802. doi: 10.1038/s41565-026-02174-5 (PMC13293870; doi:10.1038/s41565-026-02174-5)
Supplement: Supplementary file 2 — Peer Review File [file 41565_2026_2174_MOESM2_ESM.pdf]

nature portfolio

Peer Review File

Tunable Polaritonic Topologies Generated by Nonlocal Photonic Modes

Corresponding Author: Professor Andreas Tittl

This file contains all reviewer reports in order by version, followed by all author rebuttals in order by version.

Version 0:

Reviewer comments:

Reviewer #1

(Comments for the Author)
This paper proposes and experimentally validates a tunable polaritonic topology platform based on nonlocal quasi-bound states in the continuum (qBIC) resonances. The authors ingeniously employ dielectric metasurfaces to excite hyperbolic phonon polaritons (HPhPs) in hexagonal boron nitride (hBN), achieving for the first time in experiments the dynamic and continuous size tuning of polaritonic skyrmion lattices (with characteristic sizes down to λ/25), thereby eliminating the dependence on fixed wavelength-compensating structures. Furthermore, the authors demonstrate the remarkable versatility of this platform by generating kπ-twist skyrmions and meron lattices on a single chip simply by varying the resonator geometry. This study is conceptually innovative, overcoming the bottleneck of traditional topological photonic structures being fixed once fabricated, and opening a promising technological pathway for future programmable topological photonic devices, high-density optical information processing, and on-chip topological optical computing. However, I still have several points that require clarification or additional explanation from the authors:

1. Lines 100-101 state: "…By covering each resonator with hBN (Fig. 1d) and tailoring the resonance to lie within the in-plane RS-band of hBN (Fig. 1e) …". What is the specific physical meaning of "in-plane RS-band" in this context? Could the authors provide a more detailed explanation of its physical significance?
2. Lines 114-115 state: "…By tuning the excitation frequency within the Reststrahlen band of hBN…". Please explain the physical meaning of hBN's "Reststrahlen band" and its critical role in this study.
3. Lines 145-146 state: "…out-of-plane amplitude |Ez| and phase φ(Ez) can be extracted through pseudo-heterodyne (PsHet) detection…". Could the authors briefly describe the working principle of "pseudo-heterodyne detection"?
4. Lines 175-176 mention "...significantly reduce the spatial footprint". In the specific context of this research, what is the main consideration or necessity for reducing the "spatial footprint"?
5. In Figure 3 and related discussions, the size range of skyrmions is represented using λHPhP. Why is this parameter chosen? Furthermore, what is the physical reason behind the discrepancy between λHPhP and the excitation wavelength?
6. This study employs a qBIC design with a relatively low Q-factor to achieve a broader tuning range. Is the HPhP excitation efficiency directly related to the Q-factor of the qBIC? Would using a higher Q-factor (more strongly localized) qBIC significantly affect the generation or properties of the skyrmion?
7. Figure 4 shows that the sum of skyrmion numbers across seven skyrmion lattices is seven. If λHPhP is further reduced, is it possible to induce a higher skyrmion number on a single resonator? What factors determine the theoretical upper limit?
8. In Figure 4b, the legends for the red circular and red triangular data points are both solid and the same color, making them visually difficult to distinguish. It is suggested to modify them to a more easily distinguishable style (different colors or fill patterns).
9. The generation of skyrmions via nonlocal resonance in this work relies on periodic structures, which may pose challenges for device miniaturization and multifunctional integration. To assess its integration potential, could the authors provide additional data or analysis to investigate the minimum number of periodic unit cells required to generate stable skyrmions, and whether different topological textures can be produced within the same periodic area?

Reviewer #3

(Comments for the Author)
The authors present a work on the formation and tunability of photonic topological lattices by the qBIC modes of high refractive-index dielectric hexagonal resonators on a transparent CaF_2 substrate. The primary reason of the present platform

can generate the tunable photonic topological lattices is that the modes exhibit uniform intensity and phase at each resonator edge. Although the work may enable photonic topological lattices to transition from the conventional single structure scale to the photonic chip scale, we still doubt about the innovation of the work to be published in Nature Nanotechnology. Moreover, the manuscript was written at a confusing and unclear style, which may cause misunderstandings among readers.
1. First, apart from integration, what other practical applications can be anticipated in this qBIC metasurface associated topological lattices? Because the stability of the topological lattices in the near field is the main aspect that needs to be focused on, as opposed to the topological grids in free space, which can be used for high-density communications. Of course, the tunability of the topological lattices in the near field might also be quite attractive, but what advantages does this tunability have in practical applications? For instance, as the work was present, the hyperbolic phonon polaritons (HPhPs) in hexagonal boron nitride (hBN) that interfere to create highly confined photonic skyrmion lattices with sizes down to 271 nm (λ/25). However, by reducing the size of the resonator, a stronger light localization effect can also be achieved, although the mode at this time might be topological trivial.
2. Second, the emergence of the nonlocal mode relies on strong inter-field interactions rather than individual resonators, which implies that they can only occur in large-scale arrayed metasurfaces. This will also diminish the significance of this work.
3. Third, actually, we do not agree with the declaration in abstract 'However, in contrast to free-space optical skyrmions, all existing approaches to generate polaritonic field skyrmions are fundamentally limited by a lack of dynamic tunability, thus their total topological charges remain inherently fixed after fabrication'. The polaritonic field skyrmions can be also tuned in Fourier space, i.e., the by the incident phase of incident plane, which make us doubt about the novelty of the present work. The topological lattices that are tuned by the wavelength were also shown by some authors.
4. Fourth, the authors said that '…create highly confined 27 photonic skyrmion lattices with sizes down to 271 nm (λ/25)'. This is confusing. The skyrmions or skyrmion latices have a two-dimensional distribution characteristic and cannot be evaluated using a one-dimensional length dimension.
5. In Fig. 5e to 5h, the topologies can not be regarded as meron lattices because only the texture in the central area can be identified to have a topological skyrmion number of ±0.5 (From Fig. S14) and moreover, the lattices should be periodic.
6. In Fig. 3, why the excitation wavelengths of 1517 cm-1(b), 1532 cm-1(c) and 1560 cm-1(d) have a dimension of 1/m?
7. In fact, for photonic topological lattices, the formation of the topological structure depends on the symmetry of the nanostructure or the boundary. If the symmetry of the boundary is fixed, then the topological structure cannot be altered arbitrarily.
Overall, the work might be interesting to certain readers, but the current version is not yet ready for publication.

Version 1:

Reviewer comments:

Reviewer #1

(Comments for the Author)
Since the authors have addressed all my concerns, I therefore recommend the publication of this manuscript.

Reviewer #3

(Comments for the Author)
First of all, we would like to express our gratitude for the detailed response regarding the paper " Tunable Polaritonic Topologies Generated by Nonlocal Photonic Modes". The author provided clear and appropriate responses to all of our previous comments.
In fact, from our perspective, this topic is quite interesting, just as the authors have claimed 'The generation of polaritonic topologies from a qBIC resonance opens a new fundamental pathway for studying interactions between optical topologies and matter' and 'The combination of nonlocal metasurfaces with polaritonic topologies opens a wide range of potential practical applications'.
However, after carefully reviewing the rebuttal latter and the revised manuscript, the main and sole issue we focused on was: What new insights are brought about when nonlocal photonic modes are incorporated into topological optics?
We believe this is the key innovation point of this article, which may enable it to be published in Nature Nanotechnology. Although the author provided numerous sentences in the rebuttal latter, as we pointed out in our previous comments, particularly the topic 'The generation of polaritonic topologies from a qBIC resonance opens a new fundamental pathway for studying interactions between optical topologies and matter'. Can the interactions generate novel topologies? And the also the robustness of topological state against the disturbances and fabrication tolerance? We still hope that the authors can delve deeper into the significant implications off integrating non-local metamaterials with topological optics.

Open Access This Peer Review File is licensed under a Creative Commons Attribution 4.0 International License, which permits use, sharing, adaptation, distribution and reproduction in any medium or format, as long as you give appropriate credit to the original author(s) and the source, provide a link to the Creative Commons license, and indicate if changes were made.
In cases where reviewers are anonymous, credit should be given to 'Anonymous Referee' and the source.
The images or other third party material in this Peer Review File are included in the article's Creative Commons license, unless indicated otherwise in a credit line to the material. If material is not included in the article's Creative Commons license and your intended use is not permitted by statutory regulation or exceeds the permitted use, you will need to obtain permission directly from the copyright holder.
To view a copy of this license, visit https://creativecommons.org/licenses/by/4.0/

Reviewer #1 (Remarks to the Author):

General statement:

This paper proposes and experimentally validates a tunable polaritonic topology platform based on nonlocal quasi-bound states in the continuum (qBIC) resonances. The authors ingeniously employ dielectric metasurfaces to excite hyperbolic phonon polaritons (HPhPs) in hexagonal boron nitride (hBN), achieving for the first time in experiments the dynamic and continuous size tuning of polaritonic skyrmion lattices (with characteristic sizes down to λ/25), thereby eliminating the dependence on fixed wavelength-compensating structures. Furthermore, the authors demonstrate the remarkable versatility of this platform by generating kπ-twist skyrmions and meron lattices on a single chip simply by varying the resonator geometry. This study is conceptually innovative, overcoming the bottleneck of traditional topological photonic structures being fixed once fabricated, and opening a promising technological pathway for future programmable topological photonic devices, high-density optical information processing, and on-chip topological optical computing. However, I still have several points that require clarification or additional explanation from the authors:

Response:

We thank the reviewer for the positive and encouraging assessment of our work and hope we can address all concerns adequately.

Reviewer comment:

1. Lines 100-101 state: "…By covering each resonator with hBN (Fig. 1d) and tailoring the resonance to lie within the in-plane RS-band of hBN (Fig. 1e) …". What is the specific physical meaning of "in-plane RS-band" in this context? Could the authors provide a more detailed explanation of its physical significance?

2. Lines 114-115 state: "…By tuning the excitation frequency within the Reststrahlen band of hBN…". Please explain the physical meaning of hBN's "Reststrahlen band" and its critical role in this study.

Response:

Polar materials exhibit strong interaction with electromagnetic radiation and support surface phonon polaritons in isotropic systems, as well as hyperbolic phonon polaritons in anisotropic materials. These phenomena arise from the splitting between transverse optical (TO) and longitudinal optical (LO) phonon frequencies, which originates from long-range Coulomb interactions and the macroscopic polarization field generated during longitudinal lattice vibrations.

Among these materials, hexagonal boron nitride (hBN) has emerged as one of the most widely employed phonon-polaritonic platforms. Due to its anisotropy, hBN possesses two distinct Reststrahlen bands that support hyperbolic phonon polaritons in thin films. Specifically, the in-plane Reststrahlen band ($\varepsilon_{xx} < 0$, $\varepsilon_{yy} < 0$, $\varepsilon_{zz} > 0$) spans approximately 1350–1610 cm^{-1}, while the out-of-plane Reststrahlen band ($\varepsilon_{xx} > 0$, $\varepsilon_{yy} > 0$, $\varepsilon_{zz} < 0$) lies between 770–825 cm^{-1}. In principle, the lower-frequency out-of-plane Reststrahlen band

could be accessed using a qBIC resonance engineered to operate within this spectral range. However, due to the generally weaker oscillator strength and higher losses associated with this band, experimentally observing topological lattices in this regime would likely lead to reduced signal quality and lower topological robustness.

Actions taken:

We added the following section to the Introduction to clarify the role and physical origin of the various Reststrahlen bands in hBN:

"These propagating modes arise in deeply subwavelength hBN films due to long-range Coulomb interactions and the macroscopic polarization field that leads to a spectral splitting between longitudinal (LO) and transverse optical (TO) phonons, together with the intrinsic anisotropy of hBN. The anisotropy originates from the contrast between strong in-plane covalent bonding and comparatively weak out-of-plane van der Waals (vdW) interactions between adjacent layers, which leads to strong confinement of polaritons propagating within the material."

Reviewer comment:

3. Lines 145-146 state: "…out-of-plane amplitude $|E_z|$ and phase $\phi(E_z)$ can be extracted through pseudo-heterodyne (PsHet) detection…". Could the authors briefly describe the working principle of "pseudo-heterodyne detection"?

Response:

We agree that a more thorough explanation of the working principle of pseudo-heterodyne detection (PsHet) would be beneficial for the reader.

Actions taken:

We have added the following section explaining PsHet in more detail in the Methods part under "Optical near-field measurements":

"When conducting PsHet interferometry [https://doi.org/10.1063/1.2348781], the frequency of vibration for these two mirrors (around 300 Hz) is typically much lower than the tip frequency."

"Additionally, PsHet enables the elimination of the multiplicative background contribution otherwise present in all s-SNOM experiments. The reference mirror introduces side peaks next to the main tip harmonics which do not contain the multiplicative background, thus demodulation at the side band

frequencies of sufficiently high n allows for conducting near-field measurements without any far-field contributions [https://doi.org/10.1364/OE.492213].”

Reviewer comment:

4. Lines 175-176 mention "...significantly reduce the spatial footprint".In the specific context of this research, what is the main consideration or necessity for reducing the "spatial footprint"?

Response:

The reduction of the spatial footprint is a key requirement for e.g. device miniaturization and on-chip integration in future topological photonic computing platforms. First, employing a gradient metasurface [https://doi.org/10.1038/s41565-024-01767-2], rather than a conventional pixelated design [10.1126/science.aas9768] significantly increases the achievable mode density on a given chip (27500 distinct modes on a 650x450 μm^2. This allows skyrmion lattices with unit cells of different sizes to be generated within a single metasurface, instead of being restricted to a single lattice size per metasurface pixel. Previous approaches to generate topological lattices have typically relied on large-scale single launchers, such as those based on surface plasmon polaritons [10.1126/science.aau0227], to generate near-field topological textures, with dimensions several times larger than the incident wavelength. In contrast, our approach enables multiple skyrmion lattices to be compacted into a single subwavelength unit cell. For future computing devices, this high density of topological states within a compact footprint could lead to substantial increases in computational capacity.

Second, the miniaturization of such devices is highly beneficial for future studies aiming at exploring interactions between polaritonic skyrmions and other nanoscale excitations, such as excitons in two-dimensional materials, as well as strongly confined photonic modes, such as epsilon-near-zero guided modes, as these processes occur at deeply subwavelength length scales. Furthermore, exfoliated 2D material flakes typically have lateral dimensions of only a few hundred micrometers, meaning that a reduced device footprint enables more efficient integration with these materials.

Third, an important aspect of our work is that the reduction in footprint is enabled not only by the use of gradient metasurfaces, but also by the sublinear dispersion of hBN. Such strong mode compression cannot be achieved through geometry alone, particularly when the launching structures are already deeply subwavelength. Instead, it relies on the intrinsic dispersive response of phonon-polaritonic materials. The combined use of gradient metasurfaces and strongly confined hyperbolic phonon polaritons in anisotropic media enables the unprecedented subwavelength nature of our polaritonic skyrmion lattices.

Actions taken:

We added the following sentence to the Conclusion in order to highlight these advantages:

“The gradient metasurface framework employed in our work enables the investigation of these polaritonic topologies in substantially more compact systems than is possible with conventional pixelated approaches

[10.1126/science.aas9768]. This enables the use of size-limited vdW material flakes for probing the interactions between skyrmions and other excited modes at deeply subwavelength length scales."

Reviewer comment:

5. In Figure 3 and related discussions, the size range of skyrmions is represented using λHPhP. Why is this parameter chosen? Furthermore, what is the physical reason behind the discrepancy between λHPhP and the excitation wavelength?

Response:

We thank the reviewer for pointing out this potentially confusing labeling of the skyrmion size. While the size of an individual skyrmion lattice site effectively corresponds to the wavelength of the hyperbolic phonon polariton launched by the structure, we agree that these two quantities should be clearly distinguished. In this context, the size of each lattice site can be characterized by the corner-to-corner diameter of the hexagonal unit cell (distance between two opposite corners), which we call D_{hex} and now use throughout the manuscript.

To address the second point, both surface phonon polaritons and hyperbolic phonon polaritons possess in-plane momenta that are much larger than that of the incident light [https://doi.org/10.1038/s41578-023-00620-7]. This originates from the strongly dispersive dielectric response near the transverse optical phonon frequency. As a result, the polariton dispersion yields $k_{||} > k_0$, which implies that the out-of-plane wavevector $k_z = \sqrt{k_0^2 - k_{||}^2}$ becomes imaginary. Consequently, the electromagnetic fields are evanescent in the direction normal to the surface. Because free-space photons satisfy $k_{||} < k_0$, there is a momentum mismatch, and direct excitation of these modes requires additional coupling mechanisms.

Actions taken:

Throughout the manuscript and in the figures, we now explicitly label the skyrmion unit cell size as D_{hex} instead of λ_{HPhP}.

We also added the following section to explain why the polariton wavelength is intrinsically subwavelength:

"This strong confinement arises from the polariton dispersion, which yields in-plane momenta much larger than that of free-space light. As a consequence, the out-of-plane wavevector becomes imaginary, leading to evanescent decay of the fields normal to the surface [https://doi.org/10.1038/s41578-023-00620-7]."

Reviewer comment:

6. This study employs a qBIC design with a relatively low Q-factor to achieve a broader tuning range. Is the HPhP excitation efficiency directly related to the Q-factor of the qBIC? Would using a higher Q-factor (more strongly localized) qBIC significantly affect the generation or properties of the skyrmion?

Response:

The question of how the *Q*-factor influences the generated polaritonic topologies is an important one. As the reviewer correctly notes, we intentionally chose moderate *Q*-factors to maximize the window of tunability for the diameter of individual lattice sites. Our simulations indicate that the specific choice of *Q*-factor does not significantly affect the topology formed on the resonator surface. While it is conceivable that certain applications, such as lasing, would benefit from or even require sharper resonances, we do not observe any dependence of the topological characteristics on the Q factor, nor does the Q factor seem to affect the launching efficiency of HPhPs. Within the parameter range explored here, the resulting polaritonic topology remains unchanged. We also note that if the asymmetry becomes too large, i.e. the gaps between individual resonators shrinks down to tens of nanometers, direction resonator-to-resonator coupling effects may take place that distort the resulting topology.

Actions taken:

We added a new figure in the supplementary information showing that the topological skyrmion lattice maintains stability regardless of the *Q*-factor (*Q*-factors shown are between 50-200).

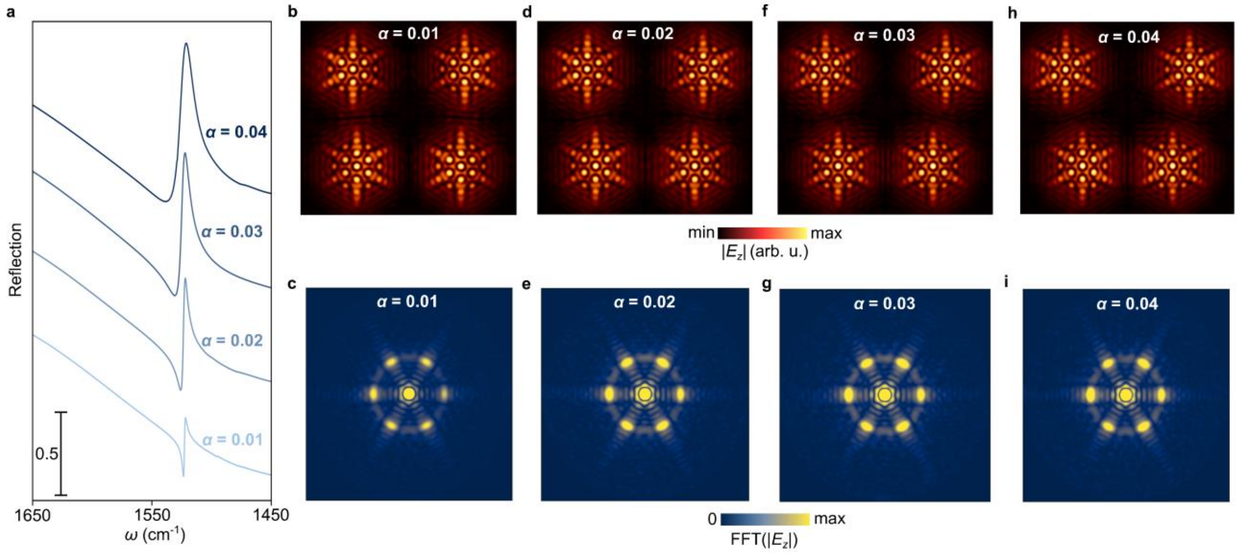

Figure S11. Polaritonic skyrmion lattices for different *Q*-factors. a Simulated reflectance spectra vs. asymmetry parameter α**.** Fourier-filtered out-of-plane near-field amplitude $|E_z|$ and absolute value of the Fourier-transformed out-of-plane electric field E_z for α = 0.01 (**b**, **c**), α = 0.02 (**d, e**), α = 0.03 (**f, g**) and α = 0.04 (**h**, **i**). All experiments were conducted on structures with α = 0.045. *Q*-factors shown are between 50-200.

Reviewer comment:

7. Figure 4 shows that the sum of skyrmion numbers across seven skyrmion lattices is seven. If λHPhP is further reduced, is it possible to induce a higher skyrmion number on a single resonator? What factors determine the theoretical upper limit?

Response:

We thank the reviewer for raising this important point. As shown in **Fig. S10**, the total skyrmion number supported by a single resonator can be tuned from values as low as 7 to numbers such as 19 or 37, and, in principle, to arbitrarily large values if losses are neglected. This scalability is enabled by the strongly sublinear dispersion of hBN, which effectively allows the wavelength of hyperbolic phonon polaritons to be compressed without a strict theoretical limit. Consequently, the total topological number is not fundamentally bounded. Additionally, by considering higher-order volume modes supported by hBN (as discussed in the Supplementary Information and **Fig. S3**) or by employing thinner flakes, the HPhP wavelength could, in principle, be further reduced by a factor of 2 or 3.

In practice, however, material losses represent a critical constraint that must be accounted for in order to experimentally observe these polaritonic topologies. To mitigate these limitations, future studies could benefit from the use of isotopically enriched hBN, which has been shown to support hyperbolic phonon polaritons with significantly extended propagation lengths [https://doi.org/10.1038/nmat5047], or from the fabrication of films with near-atomic flatness in order to reduce surface roughness that generally leads to a decrease in polariton lifetime.

Actions taken:

We added the following section to the conclusion to highlight the potential for increasing the total skyrmion number on each resonator.

*"*Another method for increasing S_T could be to employ thinner flakes or to consider higher-order hyperbolic phonon-polaritons (HPhP), with both approaches generally leading to strong compression of the HPhP wavelength. However, as HPhPs become increasingly compressed, losses play a more significant role, making the experimental observation of topological lattices challenging in standard hBN flakes. This limitation could be partially mitigated by using, for example, isotopically enriched hBN, which has been shown to support HPhPs with substantially longer propagation lengths [https://doi.org/10.1038/nmat5047]."

Reviewer comment:

8. In Figure 4b, the legends for the red circular and red triangular data points are both solid and the same color, making them visually difficult to distinguish. It is suggested to modify them to a more easily distinguishable style (different colors or fill patterns).

Response:

We agree that the points from both data sets look too similar to be properly distinguished.

Actions taken:

We changed the filling of all circular data points in **Fig. 4 b, c** to be more easily distinguishable:

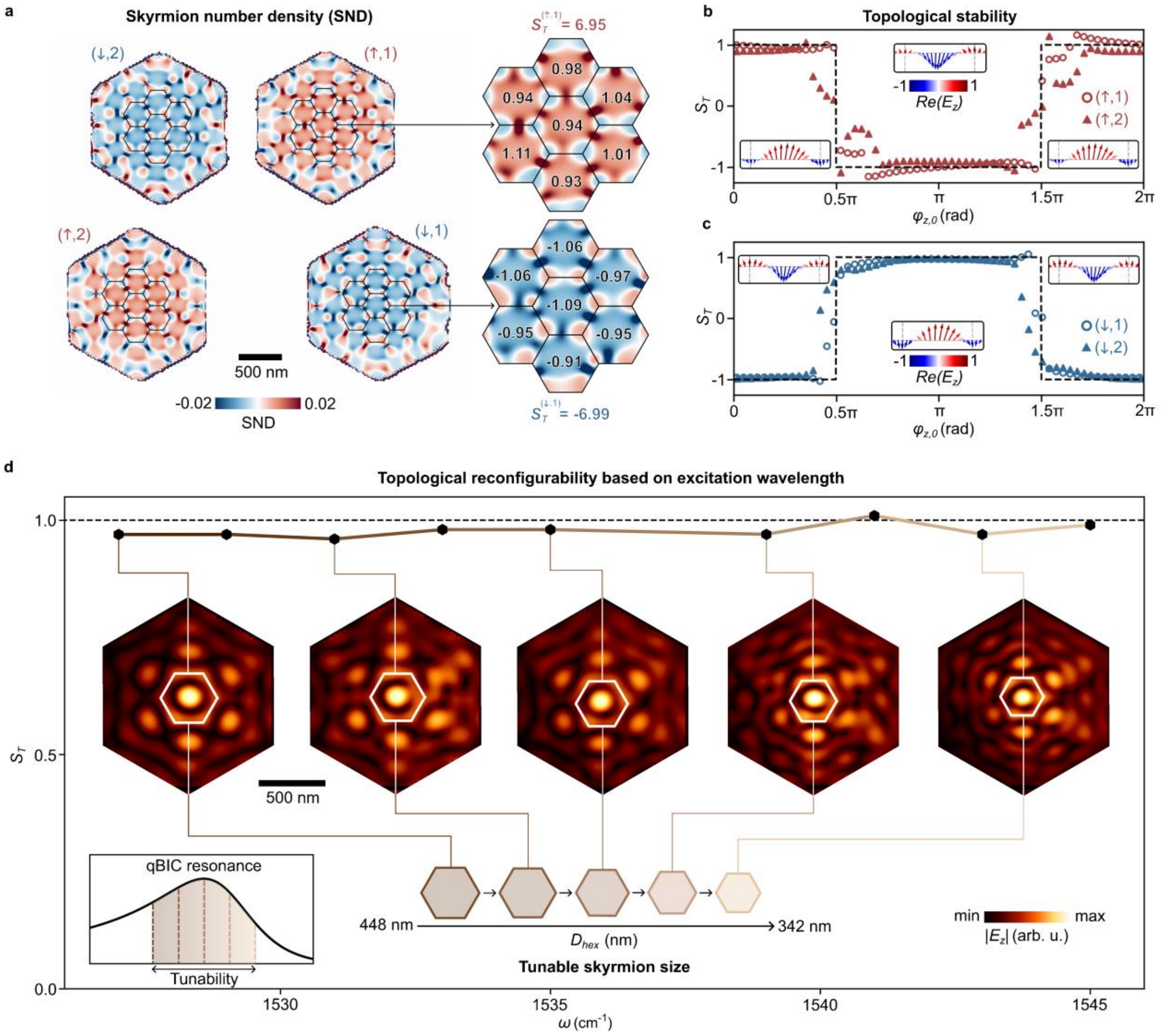

Reviewer comment:

9. The generation of skyrmions via nonlocal resonance in this work relies on periodic structures, which may pose challenges for device miniaturization and multifunctional integration. To assess its integration potential, could the authors provide additional data or analysis to investigate the minimum number of periodic unit cells required to generate stable skyrmions, and whether different topological textures can be produced within the same periodic area?

Response:

We appreciate the reviewer raising these important points concerning both the dependence of photonic mode formation on array size and the tunability between different topological textures. Below, we address these two aspects separately.

Firstly, symmetry-protected qBICs rely on periodic structures, requiring multiple unit cells in both spatial directions to establish the conditions necessary for adequate mode formation [10.1103/PhysRevLett.123.253901]. To assess the minimum number of unit cells required, it is important to consider the mode behavior in both the far field and the near field. As discussed below, the fact that photonic skyrmions reside on the surface of the resonators and therefore exist in the near field is a significant advantage, enabling a substantial reduction in the overall array size.

While in the far field, most studies conclude that more than 50 × 50 unit cells are necessary to maximize resonance modulation and *Q*-factor, a previous study by Dong et al. [https://doi.org/10.1038/s41377-021-00707-2] using electron energy-loss spectroscopy demonstrated that the photonic mode becomes fully formed after approximately **five unit cells** from the array edge. We observed a similar behavior in our study [https://doi.org/10.1002/adma.202405978] by imaging arrays of different sizes in the near field using s-SNOM, finding that an array of 5 × 5 unit cells is sufficient for proper mode formation. In addition, directional coupling effects allow for a further reduction in the number of unit cells required along the spatial direction not aligned with the incident polarization.

To study the minimum array size necessary for our mode to form, we performed an edge scan of our dielectric metasurface and found that, for our design, the maximum near-field amplitude is reached after approximately 3-4 unit cells away from the edge (see new **Fig. S6**). This indicates that only a small number of unit cells is required to establish the near-field mode. Consequently, when using hBN as an epsilon-negative material, we estimate polaritonic skyrmions can be generated with arrays as small as 7 × 7 unit cells, corresponding to an area of only approx. 35 × 35 μm^2. This corresponds to structures that are approximately five times the incident wavelength in size, as opposed to previously studied structures that generate plasmonic lattices, which were around 10-20 times the incident wavelength and were only able to generate a single skyrmion lattice.

Secondly, for a fixed non-spatially varying incident polarization, and thus a fixed incident phase, our structures do not allow direct switching between different polaritonic textures (e.g., from a skyrmion to a meron) within the same resonator. This is because the resonator boundaries uniquely determine the texture formed at its center. Nevertheless, as shown in **Fig. S10** (old **Fig. S9**), the total topological charge supported by a single resonator can be tuned through small changes in incident wavelength.

However, one could envision devices in which different textures are multiplexed within a single unit cell. This could be achieved, for example, by merging distinct resonator shapes to generate skyrmion bags [https://doi.org/10.1038/s41567-025-02873-1], or by combining multiple resonator designs within a single periodic unit cell. We illustrate this concept through simulations, demonstrating the generation of polaritonic skyrmion lattices, kπ-twist skyrmions and meron lattices, all emerging within a single periodic unit cell.

Actions taken:

Regarding the array size dependence:

We added the following supplementary section to highlight the fact that the qBIC mode forms within only a few unit cells:

"Additionally, we conduct an edge scan on the hexagon resonator metasurface (**Fig. S6**), which shows that the nonlocal mode fully forms after around 6-7 resonators, corresponding to 3-4 unit cells, suggesting that only a few unit cells are necessary to generate the qBIC mode in the near field, in agreement with previous studies [https://doi.org/10.1002/adma.202405978, https://doi.org/10.1038/s41377-021-00707-2]."

We also added a new **Fig. S6** that shows an edge scan and mode formation after only a few unit cells:

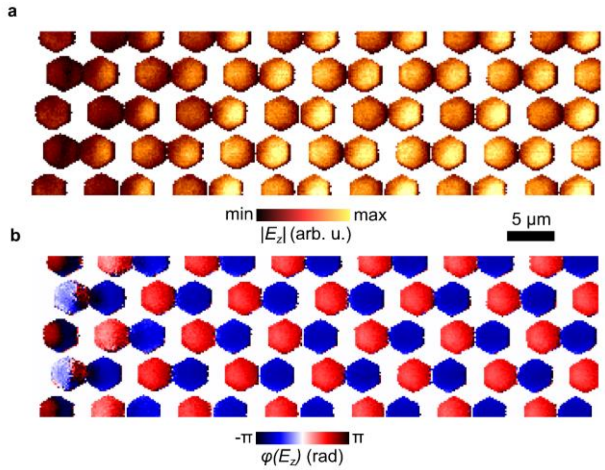

Figure S6. Edge effects of a hexagonal resonator metasurface. a Out-of-plane near-field amplitude $|E_z|$ and **b** phase $\varphi(E_z)$ of the left edge of an all-dielectric hexagonal resonator metasurface shown in Fig. 2.

Furthermore, we added the following section to the conclusion to provide an outlook on how and to what extent such a device could be miniaturized to significantly reduce the spatial footprint of the metasurface:

"In addition, the metasurfaces employed in our work could be significantly miniaturized by taking advantage of the fact that symmetry-protected qBIC modes need comparatively few unit cells to form in the near field, especially in the direction perpendicular to the polarization of the incident light [https://doi.org/10.1002/adma.202405978, https://doi.org/10.1038/s41377-021-00707-2]. In practice, when launching HPhPs using hBN as the polaritonic material, an array consisting of 7x7 unit cells (lateral size 35x35 μm^2) should be enough to generate robust skyrmion lattices within the central unit cells."

Regarding the tunability between different topological textures:

We added the following sentence to the conclusion:

"While our platform does not allow for tuning between topological lattices with different winding numbers, such as between skyrmion and meron lattices, the photonic mode employed in our work is

robust against variations of the shape of individual resonators. This stability can be leveraged to combine resonators with multiple shapes into the metasurface array to generate multiple quasi-arbitrary structured topologies with different topological properties inside each unit cell (**Fig. S20**).”

As well as a new Figure (**Fig. S20**) showing simulations of a multiplexed unit cell:

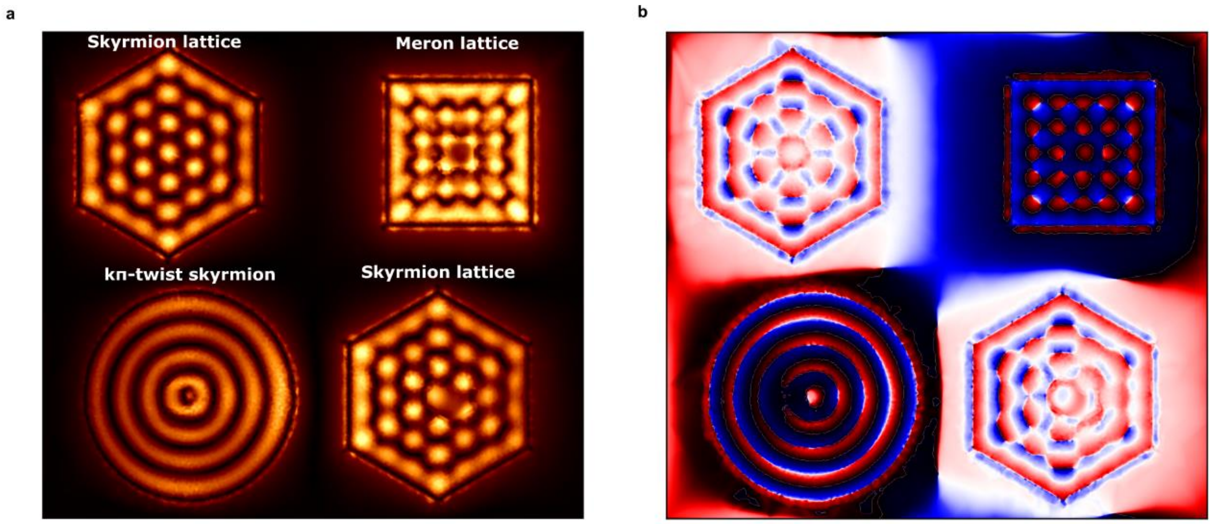

Figure S20. Simulations of a multiplexed unit cell. a Simulated out-of-plane near-field amplitude $|E_z|$ and **b** phase $\varphi(E_z)$ of a periodic array consisting of a unit cell with two hexagonal resonators (generates skyrmion lattice), a square resonator (generates meron lattice) and a disk (generates kπ-twist skyrmion).

Reviewer #3 (Remarks to the Author):

General statement:

The authors present a work on the formation and tunability of photonic topological lattices by the qBIC modes of high refractive-index dielectric hexagonal resonators on a transparent CaF2 substrate. The primary reason of the present platform can generate the tunable photonic topological lattices is that the modes exhibit uniform intensity and phase at each resonator edge. Although the work may enable photonic topological lattices to transition from the conventional single structure scale to the photonic chip scale, we still doubt about the innovation of the work to be published in Nature Nanotechnology. Moreover, the manuscript was written at a confusing and unclear style, which may cause misunderstandings among readers.

Response:

We thank the reviewer for carefully evaluating our work and hope we can address all concerns adequately.

Reviewer comment:

1. First, apart from integration, what other practical applications can be anticipated in this qBIC metasurface associated topological lattices? Because the stability of the topological lattices in the near field is the main aspect that needs to be focused on, as opposed to the topological grids in free space, which can be used for high-density communications. Of course, the tunability of the topological lattices in the near field might also be quite attractive, but what advantages does this tunability have in practical applications? For instance, as the work was present, the hyperbolic phonon polaritons (HPhPs) in hexagonal boron nitride (hBN) that interfere to create highly confined photonic skyrmion lattices with sizes down to 271 nm (λ/25). However, by reducing the size of the resonator, a stronger light localization effect can also be achieved, although the mode at this time might be topological trivial.

Response:

We thank the reviewer for raising these important points. To address the potential impact and applicability of our platform, we would like to highlight two key aspects that, in our view, distinguish it from existing approaches:

1. The generation of polaritonic topologies from a qBIC resonance opens a new fundamental pathway for studying interactions between optical topologies and matter. While previous approaches for the generation of polaritonic topological lattices relied on isolated coupling structures, our method launches propagating polariton modes through strongly localized fields originating from a nonlocal mode. This enables our metasurface to serve as a powerful platform for the fundamental study of topological phenomena at the nanoscale. For instance, bound states in the continuum can be exploited to engineer the phase with which polaritons are launched from specific boundaries without the need for additional optical components such as waveplates, potentially enabling the study of novel topologies, such as spin

skyrmion/meron lattices or polaritonic vortices, that were beyond the scope of our work, but will inspire and form the foundation of future studies. Moreover, qBIC metasurfaces provide unprecedented control over both the Q-factor and resonance position. How polaritonic topologies, and particularly polaritonic skyrmions, interact with ultrasharp resonances remains an open and intriguing question which could be of great interest for e.g. lasing and strong coupling processes.

2. The combination of nonlocal metasurfaces with polaritonic topologies opens a wide range of potential practical applications, such as on-chip metrology or refractive-index sensing, both of which benefiting from the excellent stability and deeply subwavelength features of our skyrmion lattices. For the latter, coating the metasurface with a material of unknown refractive index would compress the skyrmionic lattice pattern, enabling analytical determination of the refractive index of the coating. Since hyperbolic phonon polaritons, and therefore our polaritonic skyrmion lattices, are both tunable and highly sensitive to surface perturbations due to their evanescent out-of-plane character, our platform could offer significantly enhanced sensitivity compared to previous approaches. Furthermore, our platform could be used to explore mode hybridization with 2D materials. Exfoliated 2D material flakes typically have lateral dimensions of only a few hundred micrometers, meaning that the small footprint of our devices enables more efficient integration with these materials. Finally, as demonstrated in prior work [https://doi.org/10.1021/acs.nanolett.9b01343], skyrmion lattices and related topologies generate strong optical forces. These could be exploited to engineer trapping potentials on each resonator of our gradient metasurface, potentially enabling particle trapping, sorting, and detection, for example with the goal of monitoring microplastics in bodies of water.

Actions taken:

We added a section to the Conclusion explaining how our platform may be used for the fundamental study of optical topologies:

“By driving polaritonic topologies via qBIC resonances, our platform enables the fundamental study of different topological properties that arise through near-field phase engineering, such as spin in skyrmion lattices or orbital angular momentum in polaritonic vortices.”

We also added another section going into detail about the potential application of our tunable skyrmion lattices:

“Enabled via the highly fluctuating fields of skyrmion lattices, potential applications include localized lasing by combining our structure with an active gain medium, as well as refractive index sensing and on-chip metrology. Furthermore, skyrmion lattices and related topologies, such as Kagome lattices, can also be

used for particle trapping, sorting, and detection [https://doi.org/10.1021/acs.nanolett.9b01343], of e.g. microplastics in water."

Reviewer comment:

2. Second, the emergence of the nonlocal mode relies on strong inter-field interactions rather than individual resonators, which implies that they can only occur in large-scale arrayed metasurfaces. This will also diminish the significance of this work.

Response:

We appreciate the reviewer bringing up the dependence of the photonic mode formation on array size. *Since Reviewer 1 has asked a similar question regarding the array size effects and miniaturization potential of our platform (Comment #9), we reproduce parts of the answer given to Reviewer 1 below.*

There are important differences between near-and far-field properties when it comes to the formation of nonlocal photonic modes, since it is, in our case, the near fields that determine the formation of topological lattices. Symmetry-protected qBICs rely on periodic structures, requiring multiple unit cells in both spatial directions to establish the conditions necessary for adequate mode formation [10.1103/PhysRevLett.123.253901]. To assess the minimum number of unit cells required, it is important to consider the mode behavior in both the far field and the near field. As discussed below, the fact that photonic skyrmions reside on the surface of the resonators and therefore exist in the near field is a significant advantage, enabling a substantial reduction in the overall array size.

While in the far field, most studies conclude that more than 50 × 50 unit cells are necessary to maximize resonance modulation and *Q*-factor, a previous study by Dong et al. [https://doi.org/10.1038/s41377-021-00707-2] using electron energy-loss spectroscopy demonstrated that the photonic mode becomes fully formed after approximately **five unit cells** from the array edge. We observed a similar behavior in our study [https://doi.org/10.1002/adma.202405978] by imaging arrays of different sizes in the near field using s-SNOM, finding that an array of 5 × 5 unit cells is sufficient for proper mode formation. In addition, directional coupling effects allow for a further reduction in the number of unit cells required along the spatial direction not aligned with the incident polarization.

To study the minimum array size necessary for our mode to form, we performed an edge scan of our dielectric metasurface and found that, for our design, the maximum near-field amplitude is reached after approximately 3-4 unit cells away from the edge (see new **Fig. S6**). This indicates that only a small number of unit cells is required to establish the near-field mode. Consequently, when using hBN as an epsilon-negative material, we estimate polaritonic skyrmions can be generated with arrays as small as 7 × 7 unit cells, corresponding to an area of only approx. 35 × 35 μm^2. This corresponds to structures that are approximately five times the incident wavelength in size, as opposed to previously studied structures that generate plasmonic lattices, which were around 10-20 times the incident wavelength and were only able to generate a single skyrmion lattice.

Actions taken:

We added the following supplementary section to highlight the fact that the qBIC mode forms within only a few unit cells:

"Additionally, we conduct an edge scan on the hexagon resonator metasurface (**Fig. S6**), which shows that the nonlocal mode fully forms after around 6-7 resonators, corresponding to 3-4 unit cells, suggesting that only a few unit cells are necessary to generate the qBIC mode in the near field, in agreement with previous studies [https://doi.org/10.1002/adma.202405978, https://doi.org/10.1038/s41377-021-00707-2]."

We also added a new **Fig. S6** that shows an edge scan and mode formation after only a few unit cells:

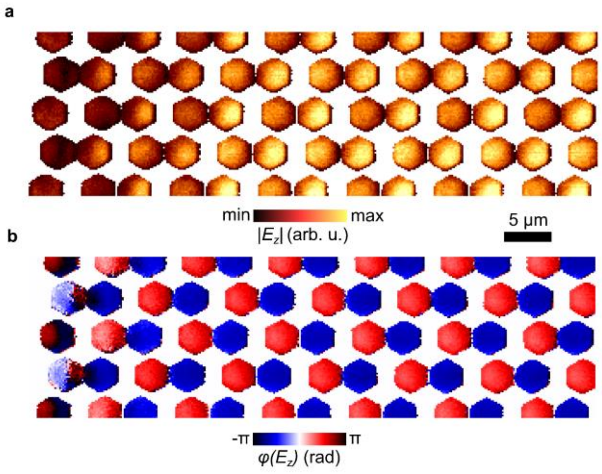

Figure S6. Edge effects of a hexagonal resonator metasurface. a Out-of-plane near-field amplitude $|E_z|$ and **b** phase $\varphi(E_z)$ of the left edge of an all-dielectric hexagonal resonator metasurface shown in Fig. 2.

Furthermore, we added the following section to the conclusion to provide an outlook on how and to what extent such a device could be miniaturized to significantly reduce the spatial footprint of the metasurface:

"In addition, the metasurfaces employed in our work could be significantly miniaturized by taking advantage of the fact that symmetry-protected qBIC modes need comparatively few unit cells to form in the near field, especially in the direction perpendicular to the polarization of the incident light [https://doi.org/10.1002/adma.202405978, https://doi.org/10.1038/s41377-021-00707-2]. In practice, when launching HPhPs using hBN as the polaritonic material, an array consisting of 7x7 unit cells (lateral size 35x35 μm^2) should be enough to generate robust skyrmion lattices within the central unit cells."

Reviewer comment:

3. Third, actually, we do not agree with the declaration in abstract 'However, in contrast to free-space optical skyrmions, all existing approaches to generate polaritonic field skyrmions are fundamentally limited by a lack of dynamic tunability, thus their total topological charges remain inherently fixed after

fabrication'. The polaritonic field skyrmions can be also tuned in Fourier space, i.e., the by the incident phase of incident plane, which make us doubt about the novelty of the present work. The topological lattices that are tuned by the wavelength were also shown by some authors.

Response:

We agree with the reviewer that the claim made in the abstract regarding the fundamental limitations of current approaches should have been qualified more precisely. For example, work by Wang *et al.* [https://doi.org/10.1103/PhysRevB.109.195434] has demonstrated through simulations that the diameter of individual plasmonic lattice sites in graphene can be tuned via the excitation wavelength when using radially polarized light. We would like to emphasize that our work aims to solve a crucial limitation of existing approaches that fundamentally limits the integration of polaritonic topologies into photonic devices: scalability.

Approaches based on structured light and spatially varying phase profiles can indeed produce the desired polaritonic topologies, but they can generally only be applied to isolated structures, unless the experimental setup becomes prohibitively complex. As a result, such topologies are often limited to a singular launcher structure per excitation beam. Moreover, the excitation of topologies within a single structure via spatially varying polarization often demands complex alignment procedures to avoid asymmetric excitation and distortion of the resulting topology, and the launcher structures themselves are usually much larger than the incident wavelength, as is the case for previously studied plasmonic skyrmion lattices [10.1126/science.aau0227].

Our platform addresses these limitations in a novel manner. First, it *relies solely on linearly polarized illumination, resulting in a spatially uniform polarization that can excite topological states over large areas*. This capability enables the generation of spatially encoded polaritonic topologies using a single excitation source, thus opening a pathway towards the adaptation of polaritonic topologies for e.g. sensing and on-chip metrology. Second, beyond standard optical components such as linear polarizers, our metasurface does not require additional elements placed in the beam path, such as spatial light modulators, which complicate alignment, render the beam path bulky, and can be particularly challenging to obtain in the mid-infrared spectral range. This represents a substantial advantage when considering future technological applications. Finally, because our polaritonic skyrmions are not generated directly by the incident beam but instead emerge from the out-of-plane fields of the qBIC metasurface, precise alignment is not required. Instead, our platform generates near-field topologies through simple illumination of the resonator array, which significantly simplifies experimental implementation and facilitates integration into practical technological applications.

Actions taken:

We changed the Abstract to more accurately reflect the novelty of our present study:

"However, in contrast to free-space optical skyrmions, all existing approaches to generate polaritonic field skyrmions are ~~fundamentally~~ limited by a lack of dynamic tunability. ~~, thus~~ In general, without engineering

the phase of the incident light, both their lattice site diameter and total topological charges remain inherently fixed after fabrication."

We also added the following section to the conclusion to highlight the advantages of our platform:

"As opposed to previous approaches using radially polarized light to generate polaritonic skyrmions [https://doi.org/10.1103/PhysRevB.109.195434], our platform makes use of incident light with spatially uniform polarization, ensuring scalability for future topological computing devices and ease of alignment."

Reviewer comment:

4. Fourth, the authors said that '…create highly confined 27 photonic skyrmion lattices with sizes down to 271 nm (λ/25)'. This is confusing. The skyrmions or skyrmion latices have a two-dimensional distribution characteristic and cannot be evaluated using a one-dimensional length dimension.

Response:

The reviewer is correct in pointing out this potentially confusing labeling. We used the corner-to-corner diameter of an individual lattice site, which corresponds to the polariton wavelength, but we realize that calling this simply the "size" of a skyrmion or labeling it as λ_{HPhP} might confuse the reader.

Actions taken:

Throughout the manuscript, we changed the word "size" to "corner-to-corner diameter" and labeled it D_{hex} instead of λ_{HPhP} when referring to the diameter of a hexagonal unit cell (i.e. distance between two opposite corners).

Reviewer comment:

5. In Fig. 5e to 5h, the topologies can not be regarded as meron lattices because only the texture in the central area can be identified to have a topological skyrmion number of ±0.5 (From **Fig. S14**) and moreover, the lattices should be periodic.

Response:

We agree that a more thorough analysis of the topology emerging from the square resonator shown in **Fig. 5e** should have been provided. We took a closer look at our data and found that the measured topology can indeed be quantified as a meron lattice, albeit clearly with reduced stability, as would be expected from a topological configuration that only covers half the unit sphere. We corroborate our findings by simulating the qBIC-driven meron lattice (see new **Fig. S17**). Similarly, simulations of qBIC-driven meron lattices show winding numbers around +-0.5, but clearly less stable and consistent than those of our skyrmion lattices. The FFT reveals that the launched polaritons exhibit the correct symmetry, showing four clearly identifiable directions in momentum space from which the HPhPs are launched, close to patterns shown in earlier works on plasmonic lattices[https://doi.org/10.1063/5.0062133]. Future

studies could assess the stability of such qBIC-driven meron lattices and potentially engineer a structure that is able to produce meron lattices of higher stability. Concerning **Fig. S16** (old **Fig. S14**), the plots were made by analytically calculating the interference of multiple plane waves, mainly for visualization purposes (in the case of a meron lattice four plane waves interfere).

Actions taken:

We conducted a more careful analysis of the meron topology from **Fig. 5** in the manuscript. To study the topological invariants that emerge from our square-shaped resonators, we added a new SI Figure showing S_T calculated for simulated and experimentally measured meron lattices:

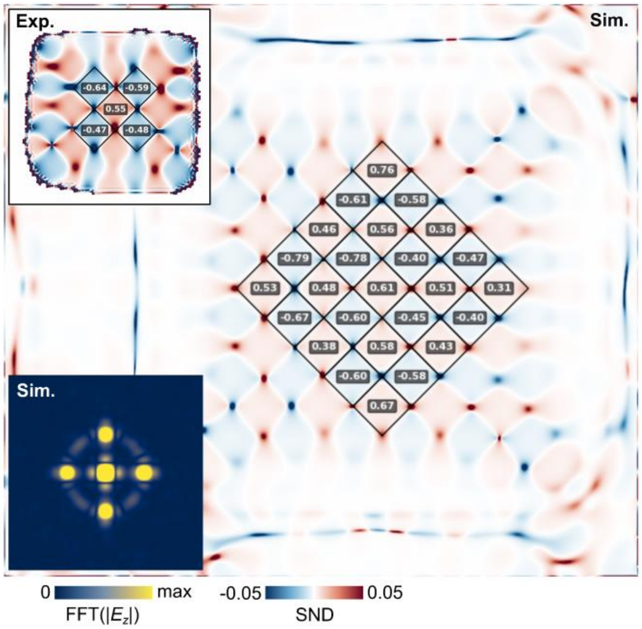

Figure S17. Stability of qBIC-driven merons. Simulated SND and FFT of $|E_z|$ and experimentally measured SND (from **Fig. 5**), with calculated S_T written within each lattice site.

Reviewer comment:

6. In Fig. 3, why the excitation wavelengths of 1517 cm-1(b), 1532 cm-1(c) and 1560 cm-1(d) have a dimension of 1/m?

Response:

The reviewer is correct in pointing out this discrepancy. We should have labeled them as excitation wavenumbers, which is a commonly used unit in mid-IR spectroscopy.

Actions taken:

We replaced wavelengths by wavenumbers in the caption of Fig. 3:

“All measurements were taken on hBN flakes with thicknesses h_{hBN} between 50 and 70 nm and excitation ~~wavelengths~~ wavenumbers of 1517 cm^{-1} (**b**), 1532 cm^{-1} (**c**) and 1560 cm^{-1} (**d**).”

as well as in other parts of the manuscript.

Reviewer comment:

7. In fact, for photonic topological lattices, the formation of the topological structure depends on the symmetry of the nanostructure or the boundary. If the symmetry of the boundary is fixed, then the topological structure cannot be altered arbitrarily.

Response:

We appreciate the reviewer asking about the tunability between different topological textures. *Since Reviewer 1 has asked a similar question regarding the array size effects and miniaturization potential of our platform (Comment #9), we reproduce parts of the answer given to Reviewer 1 below.*

In principle, for a fixed non-spatially varying incident polarization, and thus a fixed incident phase, our structures do not allow direct switching between different polaritonic textures (e.g., from a skyrmion to a meron) within the same resonator. This is because the resonator boundaries uniquely determine the texture formed at its center. Nevertheless, as shown in **Fig. S10** (old **Fig. S9**), the total topological charge supported by a single resonator can be tuned through small changes in incident wavelength.

However, one could envision devices in which different textures are multiplexed within a single unit cell. This could be achieved, for example, by merging distinct resonator shapes to generate skyrmion bags [https://doi.org/10.1038/s41567-025-02873-1], or by combining multiple resonator designs within a single periodic unit cell. We illustrate this concept through simulations, demonstrating the generation of polaritonic skyrmion lattices, kπ-twist skyrmions and meron lattices, all emerging within a single periodic unit cell.

Actions taken:

We added the following sentence to the conclusion:

“While our platform does not allow for tuning between topological lattices with different winding numbers, such as between skyrmion and meron lattices, the photonic mode employed in our work is robust against variations of the shape of individual resonators. This stability can be leveraged to combine resonators with multiple shapes into the metasurface array to generate multiple quasi-arbitrary structured topologies with different topological properties inside each unit cell (**Fig. S20**).”

As well as a new Figure (**Fig. S20**) showing simulations of a multiplexed unit cell:

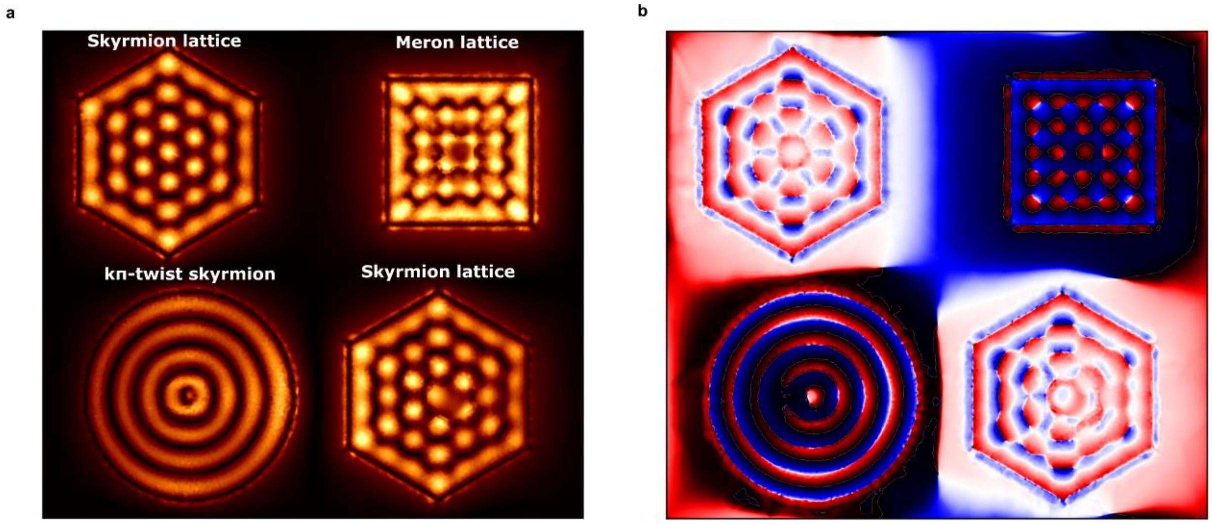

Figure S20. Simulations of a multiplexed unit cell. a Simulated out-of-plane near-field amplitude $|E_z|$ and **b** phase $\varphi(E_z)$ of a periodic array consisting of a unit cell with two hexagonal resonators (generates skyrmion lattice), a square resonator (generates meron lattice) and a disk (generates kπ-twist skyrmion).

Reviewer #3 (Remarks to the Author):

General statement:

First of all, we would like to express our gratitude for the detailed response regarding the paper " Tunable Polaritonic Topologies Generated by Nonlocal Photonic Modes". The author provided clear and appropriate responses to all of our previous comments. In fact, from our perspective, this topic is quite interesting, just as the authors have claimed ‘The generation of polaritonic topologies from a qBIC resonance opens a new fundamental pathway for studying interactions between optical topologies and matter’ and ‘The combination of nonlocal metasurfaces with polaritonic topologies opens a wide range of potential practical applications’.

However, after carefully reviewing the rebuttal latter and the revised manuscript, the main and sole issue we focused on was: What new insights are brought about when nonlocal photonic modes are incorporated into topological optics? We believe this is the key innovation point of this article, which may enable it to be published in Nature Nanotechnology. Although the author provided numerous sentences in the rebuttal latter, as we pointed out in our previous comments, particularly the topic ‘The generation of polaritonic topologies from a qBIC resonance opens a new fundamental pathway for studying interactions between optical topologies and matter’. Can the interactions generate novel topologies? And the also the robustness of topological state against the disturbances and fabrication tolerance? We still hope that the authors can delve deeper into the significant implications off integrating non-local metamaterials with topological optics.

Response:

We thank the reviewer for the positive assessment of our revised work and for raising the important question regarding the implications and potential impact of our platform for future studies of optical topologies. We address this point in two parts:

(i) Combining the physics of qBIC metasurfaces with topology introduces a fundamentally new tuning knob, enabled by the precise control over modulation and linewidth provided by the photonic mode, as well as control over the phase of the local near fields. While our work primarily explores the generation of polaritonic skyrmion lattices in a specific qBIC unit cell, a broad range of optical topologies has previously been demonstrated using free-space beams and spatial light modulators, such as Bloch-type skyrmions or higher dimensional optical hopfions. However, it is challenging to observe these topological excitations in the near-field. Our platform could lead to future studies that explore qBIC modes with various field profiles to enable the generation of more complex phase patterns, potentially giving rise to a wide variety of topological configurations beyond those demonstrated here, such as spin skyrmions, tunable skyrmion bags, and higher-order topologies.

(ii) With regard to topological robustness, our platform offers a key advantage. The resonators, with diameters of approximately 2 µm, consist of simple geometries such as hexagons, disks, or squares, and do not require additional offsets within each launcher during fabrication. In conventional approaches for generating plasmonic or phonon-polaritonic skyrmions, such offsets or gratings, which are typically on the

scale of a few hundred nanometers, could represent a major source of topological instability due to fabrication errors or local defects. By eliminating this requirement, our approach is expected to significantly enhance the robustness of the resulting topological lattices. In addition, alignment is simplified, as the structures can be excited using linearly polarized light. We hope that future studies explore the robustness of our qBIC-driven skyrmions both theoretically and experimentally.

iii) Finally, our system may be used to synergistically combine photonic modes and topological light excitations, for example by exciting with OAM light or spatially shaped light, which intrinsically contain topological charges, thus enabling more complex topological textures.

Actions taken:

We added the following part to the conclusion:

"Combining the physics of qBIC metasurfaces with topology introduces a fundamentally new tuning knob, enabled by precise control over the modulation and linewidth of the photonic mode, as well as over the phase of the local near fields. Furthermore, the omission of nanoscale offsets and grating periods may enhance the topological robustness of the generated lattices by simplifying fabrication. Our platform may also facilitate the excitation of topological charges through structured topological light fields."
